# ETS1-mediated Regulation of SOAT1 Enhances the Malignant Phenotype of Oral Squamous Cell Carcinoma and Induces Tumor-associated Macrophages M2-like Polarization

**DOI:** 10.7150/ijbs.93815

**Published:** 2024-06-11

**Authors:** Yueying Liu, Li Shen, Yi Li, Xiaoyan Sun, Lu Liang, Shiyao Jiang, Ziyun Zhang, Xingjie Tang, Yongguang Tao, Li Xie, Yiqun Jiang, Li Cong

**Affiliations:** 1 The Key Laboratory of Model Animal and Stem Cell Biology in Hunan Province, School of Medicine, Hunan Normal University, Changsha, 410013 Hunan, China.; 2 Department of Head and Neck Surgery, Hunan Cancer Hospital, Xiangya School of Medicine, Central South University, Changsha, 410013 Hunan, China.; 3 Department of Pathology, Key Laboratory of Carcinogenesis and Cancer Invasion, Ministry of Education, Xiangya Hospital, School of Basic Medicine, Central South University, Changsha, 410013 Hunan, China.

**Keywords:** Oral squamous cell carcinoma, Tumor associated macrophages, Immune infiltration, M2-like polarization, *SOAT1*, * ETS1*

## Abstract

Oral squamous cell carcinoma (OSCC) is an aggressive cancer that poses a substantial threat to human life and quality of life globally. Lipid metabolism reprogramming significantly influences tumor development, affecting not only tumor cells but also tumor-associated macrophages (TAMs) infiltration. *SOAT1*, a critical enzyme in lipid metabolism, holds high prognostic value in various cancers. This study revealed that *SOAT1* is highly expressed in OSCC tissues and positively correlated with M2 TAMs infiltration. Increased *SOAT1* expression enhanced the capabilities of cell proliferation, tumor sphere formation, migration, and invasion in OSCC cells, upregulated the SREBP1-regulated adipogenic pathway, activated the PI3K/AKT/mTOR pathway and promoted M2-like polarization of TAMs, thereby contributing to OSCC growth both *in vitro* and *in vivo*. Additionally, we explored the upstream transcription factors that regulate* SOAT1* and discovered that *ETS1* positively regulates *SOAT1* expression levels. Knockdown of *ETS1* effectively inhibited the malignant phenotype of OSCC cells, whereas restoring *SOAT1* expression significantly mitigated this suppression. Based on these findings, we suggest that *SOAT1* is regulated by* ETS1* and plays a pivotal role in the development of OSCC by facilitating lipid metabolism and M2-like polarization of TAMs. We propose that *SOAT1* is a promising target for OSCC therapy with tremendous potential.

## 1. Introduction

Head and neck squamous cell carcinoma (HNSCC) is one of the most frequent cancers worldwide 1, 2, and oral squamous cell carcinoma (OSCC) is its predominant pathological type 3, 4, with a high number of approximately 377,713 new cases worldwide in 2020 alone 5. OSCC is an aggressive cancer 6 and a major factor influencing its associated mortality is the high incidence of tumor recurrence and distant metastases 7. Although the clinical treatment of OSCC has made significant progress in recent decades, the survival rate of OSCC patients is still unsatisfactory, the prognosis is poor 4, and the 5-year survival rate is only 10% to 40% 8. OSCC seriously affects the physical and mental health as well as the quality of life of patients, causes heavy economic and medical burdens to society, and has become one of the greatest threats to the life and health of human beings.

To meet the energy and material synthesis demands for rapid multiplication, tumor cells exhibit metabolic abnormalities 9. Inducing lipotoxic effects and reducing messenger lipids by disrupting lipid metabolism homeostasis can inhibit the progression of OSCC 10. Sterol O-acyltransferase 1 (*SOAT1*) is a crucial enzyme involved in lipid metabolism that converts free cholesterol and fatty acids into cholesteryl esters (CE), which are then stored in lipid droplets (LDs). Inhibiting the activity of *SOAT1* can block the proliferation and metastasis of tumor cells 11-13. Reprogramming of lipid metabolism has been found to be closely associated with patient prognosis and immune infiltration in lung adenocarcinoma 14. Tumor-associated macrophages (TAMs) exert a crucial role in the immune response to tumors, and lipid metabolism is essential in the tumor microenvironment (TME) for promoting TAMs differentiation and function 15. However, the mechanism underlying the role of *SOAT1* in OSCC progression, particularly its connection with immune infiltration of TAMs, is currently unclear.

TAMs typically represent a substantial proportion of immune cells that infiltrate tumors 16 and are significant components of the TME 17. Immunostimulatory macrophages (M1 macrophages) and immunomodulatory macrophages (M2 macrophages) are the two phenotypes of TAMs that often polarize into different subsets depending on TME. Known as traditionally activated macrophages, M1 macrophages exhibit anti-tumor action. On the contrary, M2 macrophages, which are alternatively activated macrophages mostly found in tumors, encourage immune escape, angiogenesis, and tumor spread 18, 19. Tumor cells induce macrophage infiltration in tumor tissue and polarize macrophages to the M2 phenotype, promoting the malignant development of tumors 20. A prospective method for tumor immunotherapy involves reducing the polarization of macrophages towards the M2 phenotype 19. For example, it has been shown that the polarization of M2 TAMs in OSCC can be inhibited by targeting inhibitory eIF5A^HPU^, thereby suppressing cancer cell proliferation 21. Therefore, further exploration of the factors influencing the polarization of TAMs in OSCC is warranted and could pave the way for improved treatment of OSCC and enhanced antitumor immunity.

The ETS family of transcription factors is one of the largest families of transcriptional regulators and plays an important role in the regulation of physiological and pathological processes, with the majority of its members possessing biological properties such as the promotion of cell proliferation and differentiation and the inhibition of apoptosis 22, 23. Through bioinformatics analysis, we found that the ETS Proto-Oncogene 1 (*ETS1*) may be a transcription factor for *SOAT1*. Recent research has demonstrated that *ETS1* has a major function in the process of tumor development, and is highly expressed in most malignant tumors such as breast cancer, liver cancer, and melanoma 24. Interestingly, *ETS1* is also widely expressed in a variety of immune cell types and exhibits a crucial function in immune homeostasis 25. Ovarian cancer cells that overexpress *ETS1* can produce larger exosomes, which are more readily taken up by omental macrophages. This process can lead to the polarization of macrophages towards the M2 phenotype 26. Based on this, we believe that exploring the effect of *ETS1* on *SOAT1* is of great interest and will help provide new insights for the treatment of OSCC.

In this study, we identified that *SOAT1* promotes the malignant phenotype of OSCC through TCGA big data analysis, clinical sample validation, and cellular functional experiments. Further experiments indicated that *SOAT1* enhanced lipid metabolism in OSCC cells and promoted M2-like polarization in TAMs. Animal experiments confirmed that *SOAT1* accelerated the growth of OSCC and promoted the infiltration of M2 macrophages in transplant tumors. Additionally, we found that *ETS1* positively regulates the expression of *SOAT1* and that the *ETS1/SOAT1* facilitates the aggressive phenotype of OSCC. These findings illustrate the enormous potential of *SOAT1* as a viable target for the treatment of OSCC. Our discoveries may offer guidance for implementing immunotherapy and metabolic therapy in OSCC and supporting its clinical treatment.

## 2. Materials and methods

### 2.1. Patient data acquisition

The clinical follow-up information and gene expression profiles of HNSCC patients were obtained from The Cancer Genome Atlas (TCGA) database (https://portal.gdc.cancer.gov/), from which samples were screened for oral cancer sites, including the alveolar ridge, tongue root, buccal mucosa, oral floor, hard palate, oral cavity and tongue. There were 330 OSCC samples and 32 normal controls.

### 2.2. Immune infiltration and immunocorrelation analysis of *SOAT1*

The immune cell enrichment score was calculated using the CIBERSORT 27 algorithm, and the lollipop plot shows the correlation between *SOAT1* and 22 types of immune cells. Heatmaps and scatter plots of *SOAT1* correlation with immunoregulatory genes were displayed using the R package "ggplot2". Kaplan-Meier (KM) survival curves are utilized to estimate the probability of patient survival over time and were plotted using the R packages "survival" and "survivor".

### 2.3. Prediction of transcription factors and their binding sites

JASPAR (https://jaspar.genereg.net/) and PROMO (https://alggen.lsi.upc.es/cgi-bin/promo_v3/promo/promoinit.cgi?dirDB=TF_8.3) are two bioinformatics tools used to predict transcription factors and their binding sites in gene regulation. They provide insights into the regulatory networks of gene expression by analyzing specific patterns in genomic sequences. We used JASPAR and PROMO to predict possible transcription factors that bind to the *SOAT1* promoter region (-2000/+100 bp of the transcription start site). The binding site of *ETS1* in the *SOAT1* promoter region was predicted using JASPAR and ChIP-Atlas (http://chip-atlas.org/) was utilized to visualize the relevant binding information from the ChIP-seq dataset. The R package "ggplot2" was used for Venn diagrams to identify the intersecting transcription factors in the two databases. Combining survival time, survival status, and gene expression data, the R package "survival" was used to assess the prognostic significance of genes. A heatmap of expression correlation between intersecting transcription factors and *SOAT1* was completed by the R package "ComplexHeatmap".

### 2.4. Functional enrichment analysis

Differently expressed genes (DEGs) in the high and low expression groups were identified using the R package "limma", with the median *SOAT1* expression as the grouping criterion. The filtering criterion for DEGs was |Fold change| > 1.5, *p* < 0.05. Using the R package "clusterProfiler", DEGs were analyzed with the Kyoto Encyclopedia of Genes and Genomes (KEGG) to investigate SOAT1-related pathways.

### 2.5. Cell culture and plasmids

The human acute monocytic leukemia cell line THP-1 was acquired from the American Type Culture Collection. The human immortalized keratinocytes (HaCaT), human OSCC cell lines Cal27, HN30, HN6 and SCC-1 were purchased from Type Culture Collection of Chinese Academy of Sciences (Shanghai, China). THP-1 was cultured in RPMI 1640 (Gibco, Carlsbad, CA, United States), cells apart from THP-1 were cultured in DMEM (Gibco, Carlsbad, CA, United States). The media was supplemented with 10% (v/v) fetal bovine serum (FBS, Gibco, Carlsbad, CA, United States) and antibiotics, and all cells were maintained at 37°C in an atmosphere of 5% CO_2_. All cell lines were inspected for mycoplasma contamination and the results were negative. Macrophages were differentiated from THP-1 cells after induction of 100 ng/mL of 12-myristate 13-acetate (PMA, Sigma, United States) for 48 h.

Human *SOAT1* and *ETS1* complement DNA (cDNA) expression vector was constructed by Public Protein/Plasmid Library (Nanjing, China) with pLVX-EF1α-IRES-Puro (catalog no. 631988; Clontech, Mountain View, CA). For shRNA knockdown experiments, the shRNA vectors specifically targeting *SOAT1*, *ETS1* and a scramble control vector were purchased from Genechem (Shanghai, China), and the oligo sequences were provided in **Table 1**. All plasmid vectors were verified via sequencing.

### 2.6. H&E staining

Paraffin tissue sections were deparaffinized with xylene (twice for 10 min each) and then placed in anhydrous ethanol (twice for 10 min each), 90% ethanol (5 min), 80% ethanol (5 min), 70% ethanol (3 min) and the tissues were washed with primary water. Subsequently, the tissues were sequentially stained with hematoxylin staining solution (3 min), differentiation solution (1 sec), eosin solution (15 sec), 95% ethanol (5 sec), anhydrous ethanol (twice for 1 min each) and xylene (twice for 2 min each). Finally, the slices were sealed with neutral resin and observed by microscope (Carl Zeiss, Suzhou, China).

### 2.7. Immunofluorescence and lipid droplet staining

Cells were cultured and processed on coverslips, washed twice with phosphate-buffered saline (PBS) and then fixed with 4% paraformaldehyde for 20 min, followed by permeabilization with 0.25% Triton X-100 for 15 min. Cells were incubated with primary antibody overnight at 4°C and then treated with fluorescently labeled secondary antibody for 1 h at 37°C. The cells were incubated with DAPI at 37°C for 5 min, incubated with a drop of anti-fluorescence quencher and blocked with neutral gum, and visualized by confocal microscopy (Leica Microsystems CMS GmbH, Mannheim, Germany).

BODIPY dyes are small molecule dyes with strong UV-absorbance and are relatively stable under various physiological conditions. BODIPY lipid droplet dyes can easily penetrate the cell membrane well into the cell interior and localize specifically on polar lipids within the cell. In this study, we used BODIPY 493/503 (Abclonal, #M9850) dye for targeted staining of LDs to detect LDs (cells: 2 μM, 30 min; tissue sections: 20μM, 20 min), and was visualized by confocal microscopy.

### 2.8. Immunohistochemistry (IHC) analysis and scoring

Tissue samples were fixed by formalin and embedded in paraffin. Slides were deparaffinized and rehydrated via successive immersion in the following solutions: xylene I (10 min), xylene II (20 min), xylene: anhydrous ethanol 1:1 mixture (3 min), 100% ethanol (3 min), 95% ethanol (3 min), 75% ethanol (3 min), 50% ethanol (3 min), 30% ethanol (3 min), double distilled water I (3 min), and double-distilled water II (3 min). The slides were then boiled in 0.01 M citrate buffer for 15 min, PBS I (5 min), PBS II (5 min), double distilled water I (3 min), double-distilled water II (3 min). The slides were incubated in 3% H_2_O_2_ in methanol for 30 min which was used to block the endogenous peroxidase activity. This blocking was done with 3% bovine serum albumin (#A8020, Solarbio) solution for 1 h at room temperature. Following incubation with primary antibody at 4°C overnight, the slides were exposed to HRP-labeled secondary antibodies for 30 min at room temperature and developed with 3,3′-diaminobenzidine system. Images of the slices were captured by the microscope, and the average optical density of the slices was calculated and quantified using Image J.

### 2.9. RNA extraction and Real-Time Quantitative PCR (RT-qPCR)

As in our previous study 28, total RNA was extracted using the Trizol reagent (R401-01, Vazyme, Nanjing, China), and cDNA was synthesized using HiScript® II Q RT SuperMix for qPCR (+gDNA wiper) (R223-01, Vazyme, Nanjing, China) according to the manufacturer's instructions. RT-qPCR was performed on the Bio-Rad CFX Connect Real-Time PCR System with MonAmp™ ChemoHS qPCR Mix (MQ00401S, Monad, Shanghai, China). β-actin was used as an internal control. The primers were purchased from Sangon (Shanghai, China), and their sequences are shown in **Table 2.**

### 2.10. Western blot

Cells were washed with pre-cooled PBS, then lysed on ice using lysate buffer (#P0013, Beyotime) with a protease inhibitor (#P1010, Beyotime) for 30 min and centrifuged at 15,000 g at 4°C for 15 min. The supernatant was collected and its protein concentration was measured with the BCA assay (#E112-01, Vazyme). The following antibodies were used for western blot: mouse monoclonal anti-human β-actin antibody (#AF7018, Affinity, 1:4000), rabbit anti-human SOAT1 antibody (#A6311, ABclonal, 1:4000), mouse anti-human ETS1 antibody (#66598-1-Ig, Proteintech, 1:4000), rabbit anti-human SREBP1 antibody (#347061, Zenbio, 1:1000), rabbit anti-human SCD1 antibody (#28678-1-AP, Proteintech, 1:2000), rabbit anti-human p-PI3K antibody (#AF3242, Affinity, 1:1000), mouse anti-human PI3K antibody (#60225-1-Ig, Proteintech, 1:1000), mouse anti-human p-AKT antibody (#66444-1-Ig, Proteintech, 1:5000), mouse anti-human AKT antibody (#YM3618, ImmunoWay, 1:1000), rabbit anti-human p-mTOR antibody (#381557, Zenbio, 1:1000), rabbit anti-human mTOR antibody (#380411, Zenbio, 1:1000), goat anti-rabbit antibody (#S0001, Affinity, 1:6000) and goat anti-mouse antibody (#S0002, Affinity, 1:6000). Then the images were visualized using a luminescence kit (Vazyme, China).

Finally, the images were exposed and photographed for preservation using a chemiluminescence system (Tanon-5200, Shanghai, China).

### 2.11. Cell proliferation assay

Cells were seeded in DMEM medium (100 μL) in 96-well plates at a density of 2000 cells/well. Cell viability was measured at 0, 24, 48 and 72 h using Cell Counting Kit-8 (CCK-8, #C0005, TargetMol). Briefly, the CCK-8 solution was added to cells (10 μL/well) and incubated for 2 h. The optical density (OD) was measured at 450 nm with a microplate reader (Synengy2, Bio-Tek, United States).

### 2.12. Tumor sphere formation assay

1000 cells/well were added to 12-well low attachment plates and cultured in serum-free DMEM/F12 medium containing 2% B27, 20 ng/mL bFGF and 20 ng/mL EGF for 7-14 days. Spheres with diameters ≥50 μm in the optical microscope can be used as our selected targets for subsequent statistical analyses.

### 2.13. Colony formation assay

The cells were inoculated into 6-well plates and cultured in medium. Two weeks later, the cells were exposed to methanol for fixation and stained with 0.1% crystal violet. Image J software was used to calculate the number of visible colonies, colony diameter ≥0.05 mm was included in the counting range.

### 2.14. Transwell assay

Transwell migration and invasion assays were conducted with 8.0 μm polycarbonate membrane. For migration experiments, 5 × 10^4^ cells were distributed in 200 μL of serum-free medium were inoculated into the upper chamber, while the lower chamber was supplemented with 800 μL of medium with 10% FBS and cultured for 24 h. For invasion assays, 5 × 10^4^ cells suspended in 200 μL of serum-free medium were inoculated into the upper chamber precoated with Matrigel (082706, ABW, China), and the lower chamber was filled with 600 μL of medium containing 10% FBS and incubated for 48 h. Cells that did not cross the membrane were removed with a cotton swab, and then the cells on the bottom side of the membrane were fixed with methanol for 15 min and stained with 0.1% crystal violet. Images were viewed with a microscope and migrating and invading cells were measured with Image J.

### 2.15. Preparation of conditioned medium and macrophage treatment

To obtain conditioned medium (CM) for OSCC cells, cells that reached 80% confluence were washed and incubated with fresh medium for 24 h. The cell cultures were collected and centrifuged at 3000 rpm for 10 min, and the supernatant was collected as CM for backup. To analyze the consequences of interfering with the expression of *SOAT1* in OSCC cells on macrophage, THP-1 cells were pretreated with 100 ng/mL PMA (Sigma) for 48 h to induce them into M0 macrophages. THP-1 derived macrophages were then washed and cultured with CM of OSCC cells for 48 h.

### 2.16. Flow cytometry

Cells in logarithmic growth phase were digested into single cell suspension, centrifuged for 5 min, repeated twice, and the supernatant was discarded. 1 × 10^6^ cells were resuspended in 100 μL of CD163 primary antibody (#A8383, ABclonal, 1:100) diluted in 5% BSA (PBS configuration), protected from light at 4°C for 30 min, and centrifuged in PBS, repeated twice, and the supernatant was discarded. Then the cells were resuspended in 100 μL of BSA-diluted fluorescence. Next, cells were resuspended in 100 μL of fluorescent secondary antibody Goat Anti-Rabbit IgG (H+L) Fluor488-conjugated (#S0018, Affinity, 1:100) diluted with BSA, protected from light at 4°C for 20 min, and then centrifuged with PBS for 5 min, repeated twice, and the supernatant was discarded. Cells were resuspended in 100 μL of 1× PBS, 5 μL of PI dye was added, and the cells were incubated for 2 min at 4°C away from light. Finally, 400 μL of 1× PBS was added. Flow cytometry analysis was performed using the flow cytometer (BD FACS Lyrics) and flow cytometry data were analysed using FlowJo software (v10.6.2, FlowJo).

### 2.17. Enzyme linked immunosorbent assay (ELISA)

The concentration of cathepsin K (CTSK) in the culture medium of OSCC cells overexpressing SOAT1 and control cells was quantified by the CTSK ELISA kit (ELK Biotechnology).

### 2.18. Chromatin immunoprecipitation (ChIP)

We prepared cells with a cell count greater than 2 × 10^6^ by fixing them with formaldehyde for 10 min at room temperature, then stopped fixing with glycine for 5 min. The precipitates were resuspended by adding lysate and sonicated to disrupt the chromatin. Antibody-protein complexes were captured using pre-conjugated Protein G (Invitrogen) 29. ChIP DNA was analyzed by qPCR using SYBR Green (Bio-Rad) in an ABI-7500 (Applied Biosystems) instrument, utilizing the primers listed in **Table 3**. The following antibodies were used: mouse control IgG (AC011, Abclonal), mouse anti-human ETS1 antibody (#66598-1-Ig, Proteintech).

### 2.19. Promoter Dual-luciferase reporter assay (DLR)

The pGL4.16 luciferase vector containing the wild-type or mutant *SOAT1* promoter was constructed. Empty plasmids (pGL4.16) and dual-luciferase plasmids containing *SOAT1* Promoter WT/Mut were transfected into HEK-293 cells and Cal27 cells with ETS1 overexpression plasmids or control plasmids, respectively. Renilla luciferase was used as the standardized reporter. 24 h after co-transfection, 100 µL of luciferase detection reagent was added to each well of a 96-well plate. The luminescence signal was then detected on a bioluminescence detector to determine the relative light units (RLU). Normalized calculation = RLU of Firefly luciferase/RLU of Renilla luciferase.

### 2.20. *In vivo* tumorigenesis assay

25 female BALB/c mice, about 4 weeks old, were purchased from Hunan SJA Laboratory Animals Ltd (China). Animal experiments were conducted under the approval of the Biomedical Research Ethics Committee of Hunan Normal University. *SOAT1*-overexpressing Cal27 or *SOAT1*-knockdown HN30 cells, along with their corresponding control cell suspensions, were subcutaneously injected 100 μL into the axilla of each nude mouse (2 ×10^6^ cells/mouse). Thereafter, the body weight of the mice was monitored every three days, and the short and long diameters of the tumors were measured every three days starting on day 9 of inoculation, with the volume calculated by the formula: tumor volume (mm^3^) = 0.5 × (short diameter)^2^ × (long diameter); until day 39 of inoculation when the nude mice were euthanized and the tumors were exfoliated, photographed, and weighed. Partial tumors were homogenized for RNA extraction, or formalin-fixed, embedded in paraffin blocks and sectioned for subsequent IHC assays.

### 2.21. Statistical analysis

Every experiment was performed at least three times. All statistical analyses were performed by R software (version 4.2.3) or GraphPad Prism 9.0. Statistical significance was defined as *p* < 0.05. Differences between groups were analyzed using the Student t-test and tests of significance for more than three samples used one-way analysis of variance (ANOVA).

## 3. Results

### 3.1. Elevated SOAT1 expression is associated with an unfavorable prognosis and M2 infiltration in OSCC

**Figure 1:**
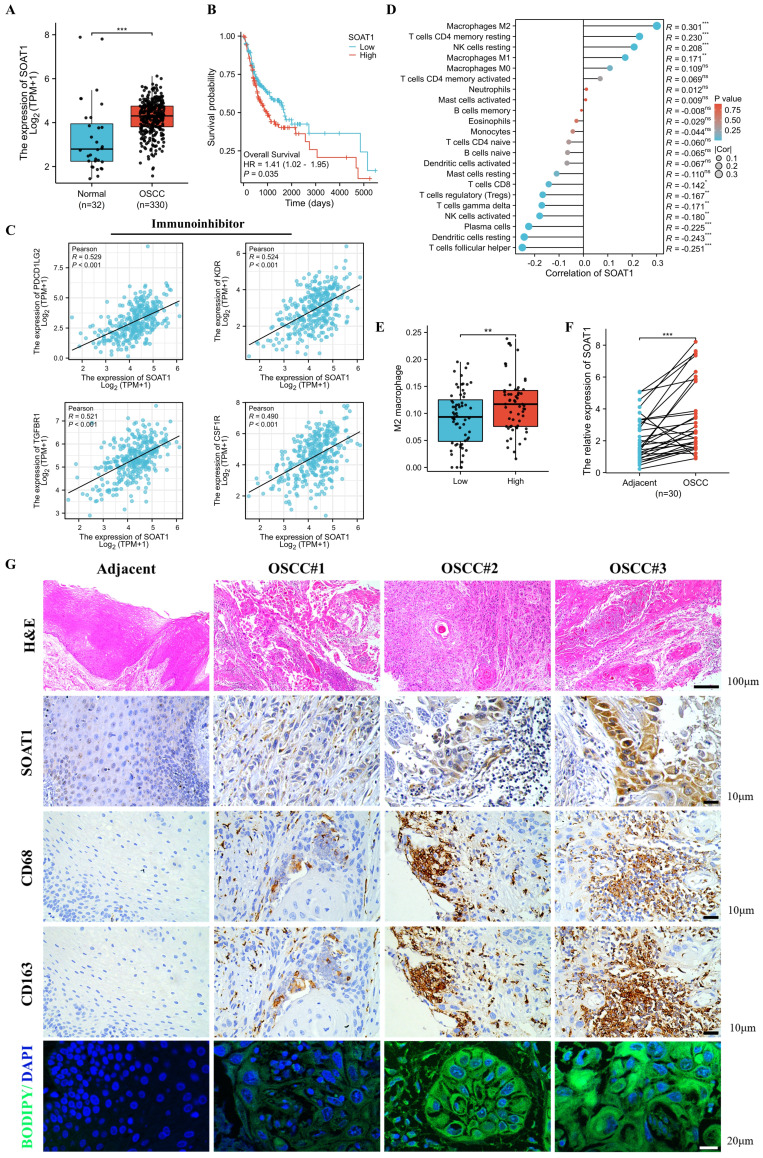
Elevated *SOAT1* expression is associated with an unfavorable prognosis and M2 infiltration in OSCC. (A) Box plot analysis of *SOAT1* expression levels between OSCC and normal tissues. (B) Kaplan-Meier curves for high and low groups of *SOAT1* expression. (C) Scatter plots of *SOAT1* correlation with immunoinhibitor genes. (D) Lollipop plot demonstrating the correlation between *SOAT1* expression and the enrichment score of immune cells calculated by the CIBERSORT algorithm. (E) Enrichment score of M2 macrophage in *SOAT1* high and low expression groups. (F) The mRNA expression of *SOAT1* in OSCC and paired adjacent tissues (n=30). (G) Human tissues from OSCC patients were stained by H&E, immunohistochemistry (IHC) or BODIPY 493/503 (green)/DAPI (blue) via using *SOAT1*, *CD68* and *CD163* antibody. Scale bar, 100 μm for H&E, 10 μm for IHC staining and 20 μm for fluorescence imaging. Bioinformatics analyses were all performed using the TCGA-OSCC dataset. The asterisks represented the statistical *p*-value (ns = no significant, *p < 0.05, **p < 0.01, ***p < 0.001).

Disordered lipid metabolism is one of the dramatically altered metabolic changes in tumors, and *SOAT1* is a critical enzyme involved in lipid metabolism. We sought to determine the degree of *SOAT1* expression in OSCC tissues. We determined that the expression of *SOAT1* is significantly higher in OSCC than in normal tissue **(Figure 1A; Figure S1A)** by analyzing data from OSCC and normal tissues in the TCGA dataset. To further explore the correlation between SOAT1 expression and the prognosis of patients with OSCC, we conducted an analysis on the relationship between SOAT1 expression and patients' overall survival (OS) as well as disease-specific survival (DSS). The results revealed that patients with high SOAT1 expression had significantly worse OS and DSS **(Figure 1B; Figure S1B)**. *SOAT1* was found to have significant and positive associations with inhibitory immunomodulatory genes *PDCD1LG2, KDR, TGFBR1* and *CSF1R,* whereas it was negatively correlated with several stimulatory immunomodulatory genes, such as *TNFRSF18* and *RAET1E* **(Figure 1C; Figure S1C-E)**. The lollipop plot revealed that among the 22 types of immune cells, M2 macrophages had the strongest correlation with *SOAT1* **(Figure 1D)**. The box plot further shows that the enrichment score of M2 macrophages was higher in the group with high expression of *SOAT1* **(Figure 1E)**. Through the bioinformatics analysis mentioned above, we discovered a strong association between *SOAT1* and poor prognosis, as well as a connection with M2 macrophages in OSCC.

We obtained 30 paired samples comprising OSCC cancer and adjacent tissues and RT-qPCR verified that *SOAT1* had higher expression in cancer compared to adjacent tissues **(Figure 1F)**. As shown in **Figure 1G**, *SOAT1* was significantly more highly expressed in OSCC tissues compared to adjacent tissues. Consistent with this finding, the content of lipid droplets was markedly more abundant in OSCC tissues.

We labeled macrophages by immunohistochemical staining of adjacent tissue sections, and cells expressing the macrophage marker *CD68* while also testing positive for the M2 macrophage marker *CD163* were considered to be M2 TAMs. The expression of *SOAT1* in OSCC coincided with the trend of M2 TAMs (CD68^+^CD163^+^) infiltration. Through TCGA big data analysis and clinical sample validation, it was found that *SOAT1* was highly expressed in OSCC and was associated with a poor prognosis. Upon further analysis of the intensity of *SOAT1* and *CD163* staining, we identified a remarkable positive correlation between *SOAT1* and M2 TAMs infiltration **(Figure S1F; r = 0.707, *p* < 0.001)**. These analyses imply that *SOAT1* has a pro-carcinogenic effect, and further studies are needed to determine its specific mechanism of action. The results suggest that focusing on the detection of lipid metabolism and M2 TAMs infiltration could be a reliable direction for research.

### 3.2. High expression of *SOAT1* promotes the malignant phenotype and lipid droplet formation of OSCC

**Figure 2:**
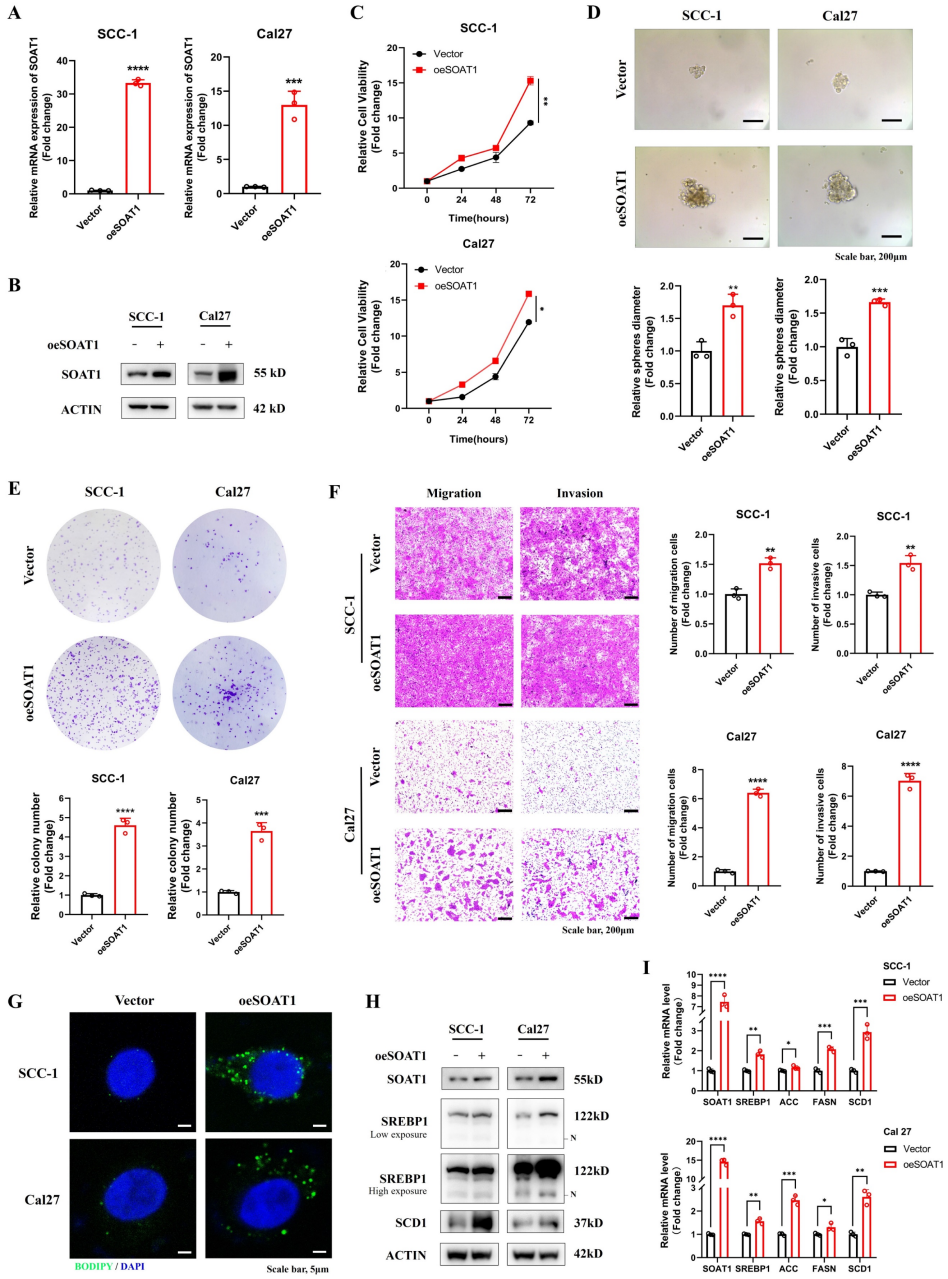
High expression of *SOAT1* promotes the malignant phenotype and lipid droplet formation of OSCC. (A) Validation of the efficacy of *SOAT1* overexpression by RT-qPCR. (B) Validation of the efficacy of *SOAT1* overexpression by western blot. (C) Cell proliferation assay of *SOAT1*-overexpressing SCC-1 cells, Cal27 cells and corresponding control cells. (D) Tumor sphere formation assay displays sphere-forming ability of cells overexpressing *SOAT1.* (E) Colony formation assay to determine the ability to form clones. (F) Transwell assay is performed to examine the effect of *SOAT1* overexpression on cell migration and invasion abilities. (G) Confocal microscopy images of OSCC cells overexpressing *SOAT1* after staining with BODIPY 493/503 (green)/DAPI (blue). Scale bar, 5 μm. (H) Western blot analysis of OSCC cells overexpressing *SOAT1*. N: N-terminal truncated form of SREBP1, its active form. (I) RT-qPCR analysis of lipid metabolism genes downstream of *SOAT1*. The asterisks represented the statistical *p*-value (*p < 0.05, **p < 0.01, ***p < 0.001, ****p < 0.0001).

**Figure 3:**
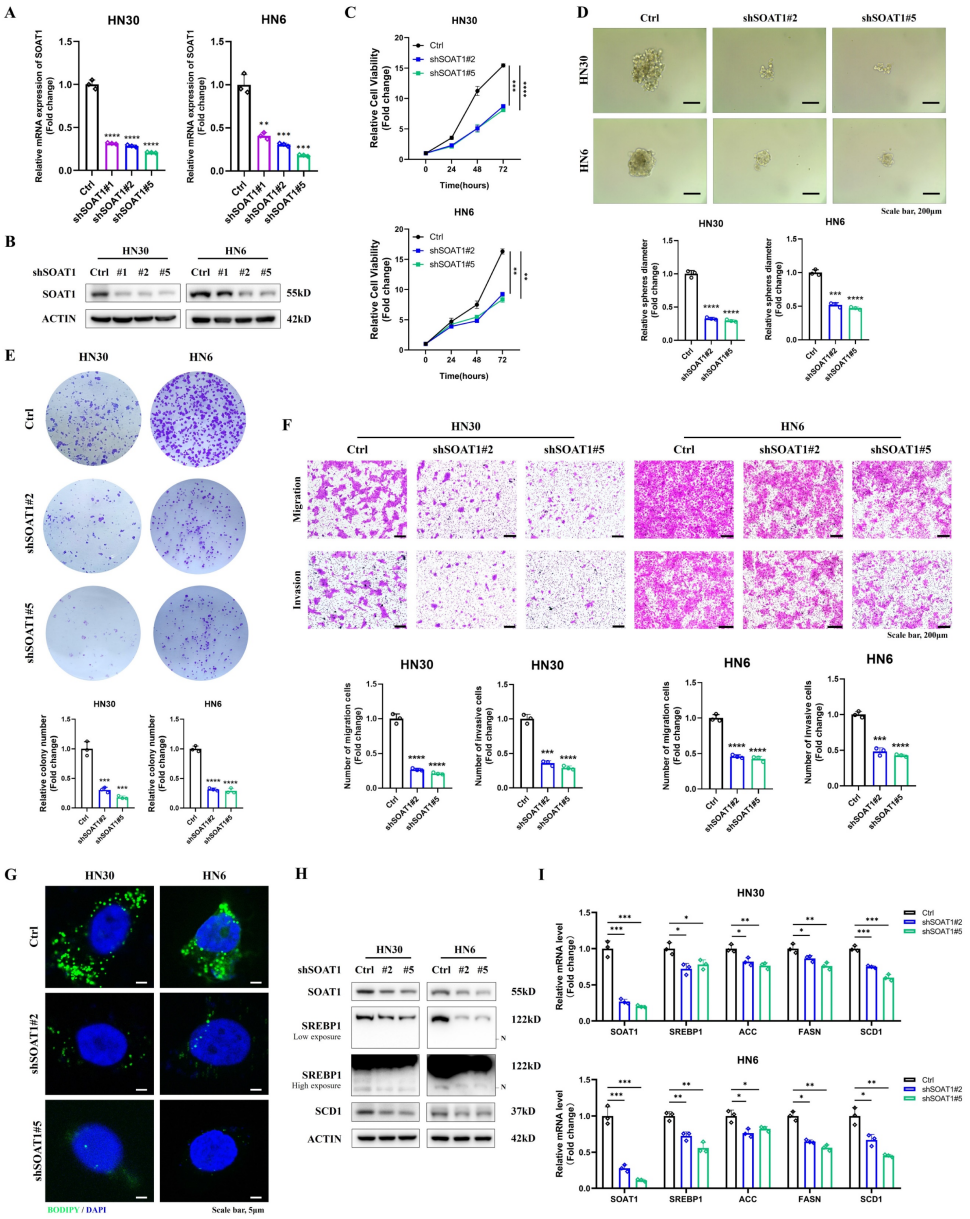
Knockdown of *SOAT1* suppresses the malignant phenotype and lipid droplet formation in OSCC. (A) Validation of the efficacy of *SOAT1* knockdown by RT-qPCR. (B) Validation of the efficacy of *SOAT1* knockdown by western blot. (C) Cell proliferation assay of *SOAT1*-knockdown HN6, HN30 cells and corresponding control cells. (D) Tumor sphere formation assay displays sphere-forming ability of cells with knockdown of *SOAT1*. (E) Colony formation assay to determine the ability to form clones. (F) Transwell assay is performed to examine the effect of *SOAT1* knockdown on cell migration and invasion abilities. (G) Confocal microscopy images of OSCC cells with knockdown of *SOAT1* after staining with BODIPY 493/503 (green)/DAPI (blue). Scale bar, 5 μm. (H) Western blot analysis of OSCC cells with *SOAT1* knockdown. N: N-terminal truncated form of SREBP1, its active form. (I) RT-qPCR analysis of lipid metabolism genes downstream of *SOAT1*. The asterisks represented the statistical *p*-value (*p < 0.05, **p < 0.01, ***p < 0.001, ****p < 0.0001).

We detected the expression of* SOAT1* in OSCC cell lines and HaCaT cell, and verified that the expression of *SOAT1* was higher in OSCC cell lines than in normal cells **(Figure S2)**. Given the relatively low expression of *SOAT1* in SCC-1 and Cal27 cells, we selected them to construct stable overexpression cell lines. In addition, due to the high expression level of *SOAT1* in HN6 and HN30 cells, these cell lines were subjected to *SOAT1* knockdown. Subsequently, validation was performed by RT-qPCR **(Figure 2A, 3A)** and western blot** (Figure 2B, 3B)**. Cell proliferation ability was examined using the CCK-8 assay, and we detected cell proliferation within 72 h. The outcome suggested that cells overexpressing *SOAT1* exhibited significantly enhanced proliferation ability compared to the control group **(Figure 2C)**. In tumor sphere formation experiments, our results suggested that in *SOAT1*-overexpressing cells, the ability to form tumor spheres was significantly increased compared to the control **(Figure 2D)**. In addition, colony formation assays indicated that* SOAT1* promoted the proliferation of OSCC cells **(Figure 2E)**. We explored the impacts of *SOAT1* overexpression on the migratory and invasive abilities of OSCC cell lines using a transwell assay. These experiments revealed that the migratory and invasive abilities of the cell lines with stable *SOAT1* overexpression were significantly improved **(Figure 2F)**. The aforementioned experiments proved that overexpression of *SOAT1* was able to promote the malignant phenotype of OSCC.

Subsequently, we investigated whether *SOAT1* was capable of affecting the storage of cholesteryl esters, which was also known as the formation of LDs, in OSCC cells. Confocal images displayed that overexpression of *SOAT1* significantly increased the formation of LDs in OSCC cells **(Figure 2G)**. We investigated whether interfering with the expression level of *SOAT1* in OSCC cells may impact SREBP activity considering it has been discovered that feedback inhibition of SREBP is caused by cholesterol esterification 13. The plots of western blot displayed that overexpression of *SOAT1* in OSCC cells significantly enhanced sterol regulatory element-binding protein-1 (*SREBP1*) activation, which was represented by an increase in the expression of N-terminal truncation of *SREBP1* **(Figure 2H)**. In addition, elevated expression of SREBP1-regulated downstream lipogenic enzymes, including acetyl-CoA carboxylase (*ACC*), fatty acid synthase (*FASN*) and stearoyl-CoA desaturase-1 (*SCD1*) was observed in cells overexpressing *SOAT1* **(Figure 2H-I)**. Overall, these findings forcefully support the idea that *SOAT1* is essential for regulating the formation of LDs and the malignant phenotype in OSCC. High expression of *SOAT1* promotes the malignant phenotype and lipid droplet formation of OSCC.

### 3.3. Knockdown of *SOAT1* suppresses the malignant phenotype and lipid droplet formation in OSCC

Combining the results of RT-qPCR and western blot validation, we selected shSOAT1#2 and shSOAT1#5, which exhibited superior knockdown effects in both HN6 and HN30 cells, for subsequent experiments **(Figure 3A-B)**. By CCK-8, tumor sphere formation, colony formation, and transwell assays, we noticed that the knockdown of *SOAT1* dramatically inhibited the proliferation, migration, and invasion abilities of OSCC cells **(Figure 3C-F)**. Subsequently, we similarly investigated the effect of *SOAT1* on lipid droplet formation. The results of lipid droplet staining displayed that knockdown of *SOAT1* greatly reduced the content of LDs in OSCC cells **(Figure 3G)**.

Western blot results suggested that the decreased expression of *SOAT1* in OSCC cells suppressed the activation of *SREBP1* and the expression of its downstream adipogenic enzymes, including *ACC, FASN*, and *SCD1* **(Figure 3H-I)**. The above outcomes suggest that the suppression of *SOAT1* hinders the malignant phenotype and formation of LDs in OSCC. The tumor-promoting impact of* SOAT1* in OSCC may be attributed to its effect on lipid metabolism.

### 3.4. *SOAT1* upregulates the PI3K/AKT/mTOR pathway

To investigate the molecular mechanisms related to *SOAT1*, we used the TCGA-OSCC dataset and divided the samples into high and low expression groups based on the median *SOAT1* expression. We screened the differentially expressed genes between these two groups **(Figure S3A)** and performed KEGG analysis on these genes. The results of the KEGG analysis showed that the PI3K/AKT pathway was significantly up-regulated **(Figure S3B)**, which was in line with existing studies that emphasize the importance of the PI3K/AKT/mTOR pathway in regulating the growth, survival and metabolism of various tumor cells 30. In particular, activation of the PI3K/AKT/mTOR pathway is closely associated with the development of OSCC 31, 32. Based on these findings, we hypothesized that the PI3K/AKT/mTOR pathway might be involved in the role of *SOAT1* in promoting OSCC development.

To test this hypothesis, we conducted western blot experiments to measure the expression levels of p-PI3K, PI3K, p-AKT, AKT, p-mTOR and mTOR in OSCC cells with overexpression and knockdown of *SOAT1*. The experimental results illustrated that the expression levels of p-PI3K, p-AKT, and p-mTOR were significantly elevated in OSCC cells overexpressing* SOAT1*, whereas the phosphorylation levels of these proteins were decreased in *SOAT1* knockdown cells **(Figure S3C)**. These results strongly suggest that *SOAT1* indeed affects the activation of the PI3K/AKT/mTOR pathway, which may be one of the mechanisms by which it promotes the development of OSCC.

### 3.5. *SOAT1* enhances the M2-like polarization of TAMs

**Figure 4:**
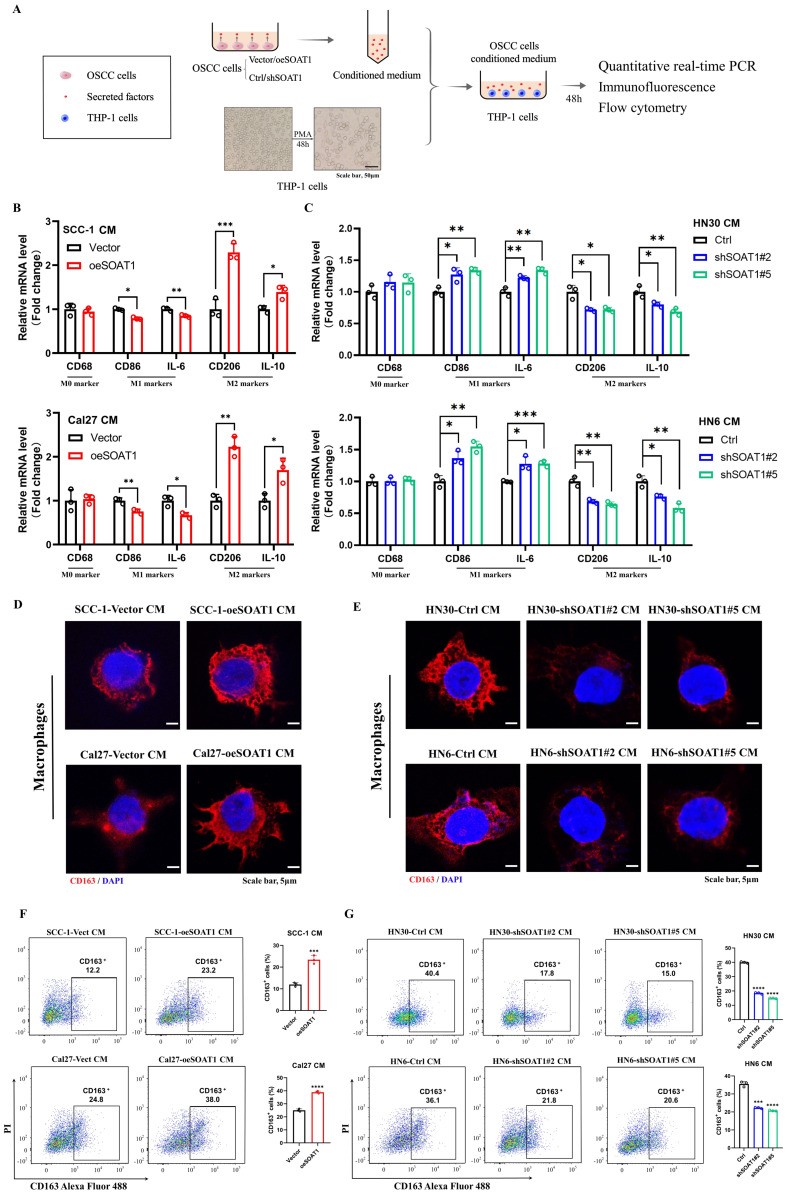
*SOAT1* enhances the M2-like polarization of TAMs. (A) Schematic diagram of macrophage treatment. (B-C) After THP-1-derived macrophages were cultured with conditioned medium (CM) for 48 h, the mRNA levels of macrophage markers were measured by RT-qPCR. (D-E) Cellular immunofluorescence assays were performed to determine the localization and variation in fluorescence intensity of CD163 in macrophages cultured with CM. (F-G) Flow cytometry to assess the expression level of CD163 in macrophages after culture in conditioned media. The asterisks represented the statistical *p*-value (*p < 0.05, **p < 0.01, ***p < 0.001, ****p < 0.0001).

The preliminary analysis had already identified a strong correlation between *SOAT1* and M2 TAMs, and we wanted to further investigate the impact of *SOAT1* expression levels in OSCC cells on TAMs polarization *in vitro*, therefore, we cultured macrophages using conditioned media (CM). **Figure 4A** illustrates the process of treating macrophages using CM for OSCC cells. We collected supernatants of OSCC cells with/without *SOAT1* overexpression or knockdown, and then incubated THP-1-derived macrophages with supernatants from different sources. The relative expression of M0 (*CD68*), M1 (*CD86, IL-6*) and M2 (*CD163, CD206, IL-10*) markers was measured after incubation of macrophages with CM for 48 h. The outcomes revealed that overexpression of *SOAT1* decreased the mRNA expression of M1 macrophage markers and increased the levels of M2 macrophage markers, and the CM of knockdown cells displayed the opposite effect **(Figure 4B-C)**. We observed the expression of the M2 macrophage marker CD163 using immunofluorescence. Macrophages cultured with CM of OSCC cells overexpressing *SOAT1* exhibited higher *CD163* fluorescence intensity, whereas and knockdown of *SOAT1* caused a decrease in the expression of *CD163* **(Figure 4D-E)**. Quantification of CD163^+^ cells using flow cytometry showed a significantly higher proportion of CD163^+^ cells in the overexpression group and a markedly fewer CD163^+^ cells in the knockdown group compared to the control group **(Figure 4F-G)**, suggesting that the level of *SOAT1* in OSCC cells can influence TAMs polarization. Specifically, we concluded that *SOAT1* in OSCC promotes the process of M2-like polarization of TAMs.

Cholesterol efflux transporter proteins *ABCA1* and *ABCG1* in macrophages 33, and the cytokines secreted by tumor cells including *CTSK* 34, *IL-10* and *CSF1* 35, have been shown to trigger the polarization of TAMs towards the M2 phenotype. We detected the genes mentioned above using RT-qPCR. The expression levels of *ABCA1* and *ABCG1* were elevated in macrophages cultured with the CM from Cal27 cells overexpressing *SOAT1* **(Figure S4A)**. In OSCC cells with overexpressed or knocked down *SOAT1*, we observed a trend of *CTSK* changes consistent with *SOAT1* **(Figure S4B-C)**. The levels of the tumor-secreted cytokine CTSK in the supernatant of OSCC cells were further measured by ELISA assay. We observed a significant increase in the levels of *CTSK* in the supernatant of *SOAT1* overexpressing cells compared to the control group **(Figure S4D)**. To validate the function of *CTSK* in promoting M2-like polarization of TAMs, we added odanacatib, a specific inhibitor of CTSK 34, to CM and incubated macrophages, with flow cytometry detecting the percentage of CD163^+^ cells. We observed a significant increase in the proportion of CD163^+^ cells in the oeSOAT1 group compared to the control group, while odanacatib treatment partially reversed the increase in the proportion of CD163^+^ cells caused by *SOAT1* overexpression **(Figure S4E)**. These data suggest that *CTSK* is indeed involved in SOAT1-induced M2 polarization of macrophages.

### 3.6. *ETS1* transcription upregulates *SOAT1* expression and influences lipid metabolism in OSCC cells

**Figure 5:**
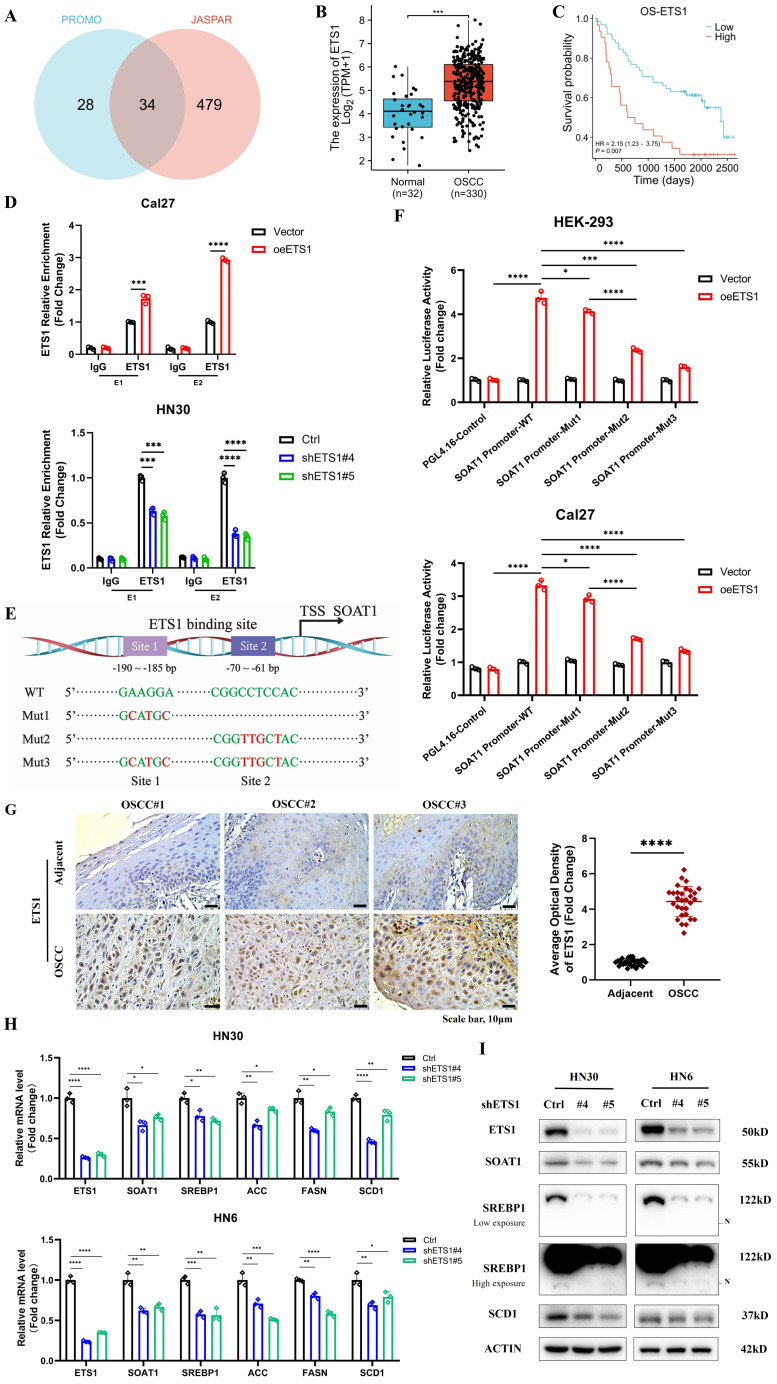
*ETS1* transcription upregulates *SOAT1* expression and influences lipid metabolism in OSCC cells. (A) Venn diagram of *SOAT1* upstream transcription factors screened by the PROMO and JASPAR databases. (B) Box plot analysis of *ETS1* expression levels between OSCC (n=330) and normal tissues (n=32). (C) Kaplan-Meier curves for high and low groups of *ETS1* expression. (D) ChIP assays are conducted to investigate the enrichment of *ETS1* in the *SOAT1* promoter region of cells that were overexpressing or knocking down *ETS1* and the corresponding control cells. (E) Schematic representation of the luciferase reporter gene of the *SOAT1* promoter and its mutant (Mut) construct. (F) Empty plasmids (pGL4.16) and dual-luciferase plasmids containing WT, Mut1, Mut2 or Mut3 of *SOAT1* promoter were transfected into HEK-293 cells and Cal27 cells with *ETS1* overexpression plasmids or control plasmids, respectively. Firefly and Renilla luciferase signals were performed for luciferase activity after 24h of transfection. TSS: Transcription start site. (G) Immunohistochemistry detects the expression of *ETS1* in OSCC tissues and adjacent tissues of cancer. (H-I) The expression levels of downstream lipid metabolism genes in OSCC cells with *ETS1* knockdown were checked using RT-qPCR and western blot. The asterisks represented the statistical *p*-value (*p < 0.05, **p < 0.01, ***p < 0.001, ****p < 0.0001).

Lipid metabolism is regulated by multiple transcription factors, and we attempted to explore the upstream transcription factors that modulate the expression of *SOAT1*, thus identifying 34 overlapping transcription factors through predictions on PROMO and JASPAR websites **(Figure 5A)**. The expression levels of the above transcription factors in OSCC tissues versus normal tissues were analyzed using TCGA-OSCC expression profiling data, revealing that 22 genes were remarkably highly expressed in tumors **(Figure S5A)**. The correlation between the 22 genes and *SOAT1* was further analyzed, among which *ETS1* showed the highest correlation with *SOAT1* at 0.79 **(Figure S5B)**, from which we hypothesized that *ETS1* is an important upstream transcription factor of *SOAT1*. We discovered that *ETS1* was highly expressed in OSCC and linked with a poor prognosis by examining TCGA-OSCC data **(Figure 5B-C)**. The cell lines with stable overexpression and knockdown of *ETS1* were constructed, and the expression of *SOAT1* was examined. The results revealed that the overexpression of *ETS1* upregulated the expression level of *SOAT1*, while the expression of *SOAT1* was suppressed when *ETS1* was knocked down **(Figure S6A)**. Combined with RT-qPCR and western blot results, shETS1#4 and shETS1#5 exhibited superior knockdown efficiencies, and we selected them for subsequent experiments.

In gene sequences, motifs are repetitive segments that act as anchors which are typically recognized and bound by transcription factors. Using the JASPAR and PROMO websites, we projected the existence of *ETS1* binding sites in the *SOAT1* promoter region and acquired motif sequences for the corresponding regions. Binding peak data of *ETS1* in the *SOAT1* promoter region from ChIP-Atlas were visualized with IGV. The results displayed that the binding peaks at the E1 (-190 ~ -185) and E2 (-70 ~ -61) sites of the *SOAT1* transcriptional start region were the most intensive **(Figure S6B)**. We further detected the level of *ETS1* enrichment at E1 and E2 sites in the *SOAT1* promoter region through a ChIP assay. The results were depicted in **Figure 5D**, *ETS1* was found to be capable of binding to both E1 and E2 sites. The enrichment was enhanced by the overexpression of *ETS1* and dramatically decreased by the knockdown of *ETS1*. Promoter dual luciferase reporter assay was performed in HEK293 and Cal27 cells to confirm whether *ETS1* transcriptionally activates the *SOAT1* promoter. Wild type and corresponding mutants were constructed **(Figure 5E)**. We found that the overexpression of *ETS1* upregulated the luciferase activity of the reporter gene, whereas mutations in both binding sites partially reduced the elevated luciferase activity, confirming the specificity of the effect **(Figure 5F)**. Furthermore, the mutation at the E2 site suppressed the elevation of luciferase activity to a greater extent compared to Mut1, suggesting that *ETS1* had a superior enrichment at the E2 site. These results demonstrate that *ETS1* acts as a transcription factor that positively regulates the transcriptional level of *SOAT1* mainly by binding to the E2 site.

Afterwards, additional experiments were conducted to confirm the regulatory effect of *ETS1* on *SOAT1* expression. Immunohistochemical staining and RT-qPCR results showed that *ETS1* was significantly more highly expressed in OSCC tissues compared with adjacent tissues **(Figure 5G; Figure S7A)**. Polyphyllin I (PPI), a small molecule extracted from the rhizome of the traditional Chinese medicine "Paris polyphylla", has been reported to have anti-cancer effects in various tumors 36, 37. Recently, PPI was found to be able to down-regulate *ETS1* expression 38. After treating Cal27 cells with various concentrations of PPI for 48 h, western blot results revealed a significant suppression of *ETS1* protein expression in Cal27 cells treated with 4 μM PPI, meanwhile, the expression of *SOAT1* was noticeably reduced **(Figure S7B)**. On the basis of *ETS1* overexpression, the cells were treated with 4 μM PPI for 48 h. At the protein level, elevated *ETS1* expression was accompanied by similarly elevated *SOAT1* expression. Compared with the unspiked group, the PPI-treated cells all had decreased levels of* ETS1* expression, and there was a consistent trend in the expression levels of *SOAT1* **(Figure S7C)**. Overexpression of* ETS1* resulted in an upregulation of *SOAT1*, while PPI inhibited the expression of *ETS1* and mitigated the elevation of *SOAT1* protein level. Furthermore, staining of adjacent tissue sections showed a strikingly strong positive correlation between *ETS1* and *SOAT1* expression **(Figure S7D-E; r = 0.737, *p* < 0.001)**. Thus, we obtained powerful evidence verifying the existence of a positive regulatory effect of *ETS1* on the expression of *SOAT1*.

The investigation of the potential impact of changes in the expression of *ETS1*, an upstream transcriptional regulator of *SOAT1*, a crucial gene involved in lipid metabolism, on lipid metabolism in OSCC cells is a topic that we anticipate exploring. Therefore, we used western blot and RT-qPCR to identify alterations in the expression of genes involved in lipid metabolism following the modulation of *ETS1* expression. The findings of this study indicate that the overexpression of *ETS1* upregulated the expression levels of the aforementioned genes involved in lipid metabolism. Conversely, the knockdown of *ETS1* led to a suppression of their expression **(Figure S7F-G; Figure 5H-I)**. Taken collectively, it can be determined that *ETS1* exerts a positive regulatory effect on the expression of *SOAT1* and interferes with lipid metabolism in OSCC cells.

### 3.7. *ETS1/SOAT1* drives the aggressive phenotype and lipid metabolism of OSCC cells

**Figure 6:**
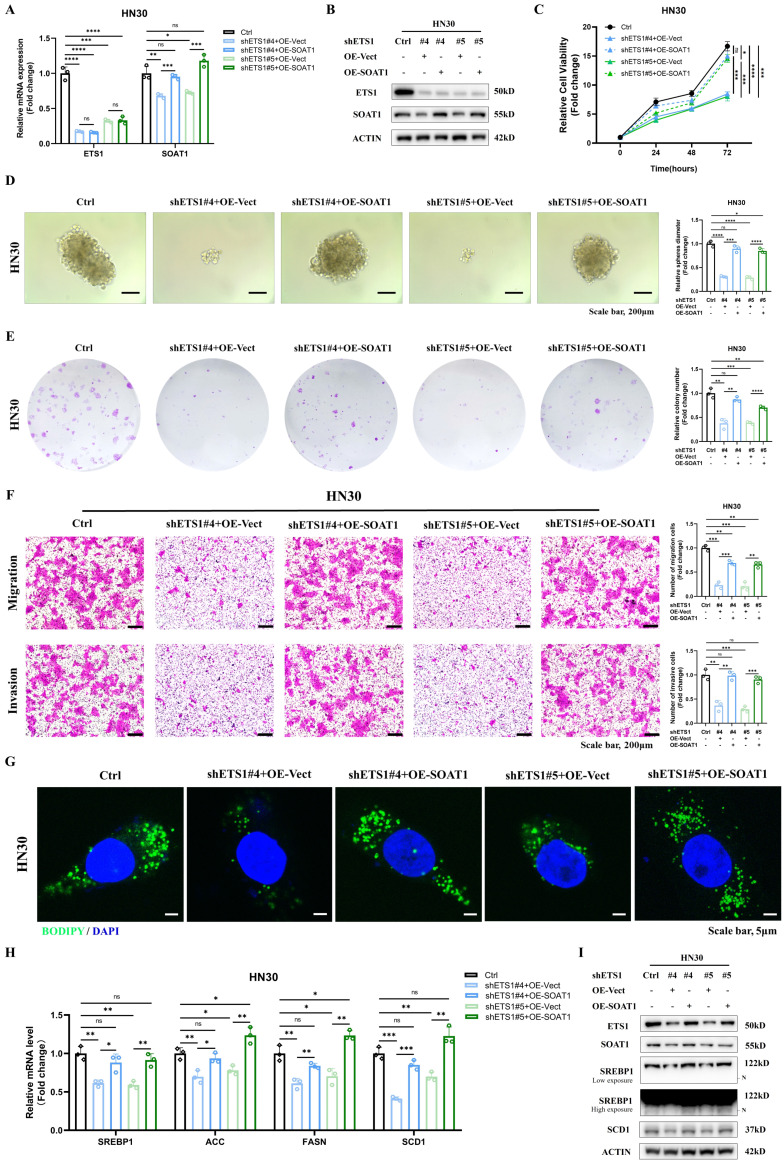
*ETS1/SOAT1* drives the aggressive phenotype and lipid metabolism of OSCC cells. (A-B) RT-qPCR and western blot assay were performed to detect the effect of *SOAT1* overexpression in *ETS1* knockdown cells. (C) CCK-8, (D) Tumor sphere formation, (E) Colony formation and (F) Transwell assay were conducted to assess the effects of *ETS1* knockdown on the proliferation, tumor sphere formation, migration and invasion of HN30 cells and the impact of *SOAT1* overexpression on the effects of HN30 Ctrl/shETS1 cells on the above-mentioned abilities was evaluated. (G) Confocal microscopy images of OSCC cells with *ETS1* knockdown and *SOAT1* overexpression after staining with BODIPY 493/503 (green)/DAPI (blue). Scale bar, 5 μm. (H) RT-qPCR analysis of lipid metabolism genes downstream of *SOAT1*. (I) Western blot analysis of lipid metabolism genes downstream of *SOAT1* in OSCC cells overexpressing *SOAT1* after knockdown of *ETS1*. N: N-terminal truncated form of SREBP1, its active form. The asterisks represented the statistical *p*-value (ns = no significant, *p < 0.05, **p < 0.01, ***p < 0.001, ****p < 0.0001).

To investigate whether *ETS1* exerts its biological function in OSCC through *SOAT1*, we conducted rescue experiments using the HN30 cell line, which exhibits superior *ETS1* knockdown effects. We knocked down *ETS1* in the HN30 cell line while transiently transfecting the *SOAT1* plasmid, which was verified using RT-qPCR and western blot **(Figure 6A-B)**. Through a series of functionalistic experiments, including CCK-8, tumor sphere formation, colony formation and transwell assays, we observed that the knockdown of ETS1 significantly suppressed the capability of OSCC cells to proliferate, form tumor spheres, migrate and invade. However, the restoration of *SOAT1* expression apparently reversed the inhibitory effect caused by *ETS1* knockdown **(Figure 6C-F)**. All of the above experiments illustrate that *ETS1* can regulate the progression of OSCC through *SOAT1*.

Afterwards, we explored whether *ETS1* regulates lipid metabolism in OSCC cells via *SOAT1*. Fluorescent staining results implied that the knockdown of *ETS1* decreased lipid droplet formation, while the recovery of *SOAT1* expression markedly increased the lipid droplet content in cells** (Figure 6G)**. The expression of *SREBP1* and its downstream lipid-forming enzymes *ACC, FASN* and *SCD1* was down-regulated in cells with knockdown of *ETS1*, while the expression level was rebounded after overexpression of* SOAT1* **(Figure 6H-I)**. Collectively, the restoration of *SOAT1* expression in *ETS1*-knockdown OSCC cells recovered the decreased proliferation, tumor sphere formation, migration, and invasion abilities, as well as the inhibitory effects on lipid metabolism-related genes resulting from *ETS1* knockdown, suggesting that *ETS1* modulates lipid metabolism through *SOAT1* and subsequently influences the progression of OSCC cells.

### 3.8. *SOAT1* augments tumor growth and facilitates M2 infiltration in xenografts

**Figure 7:**
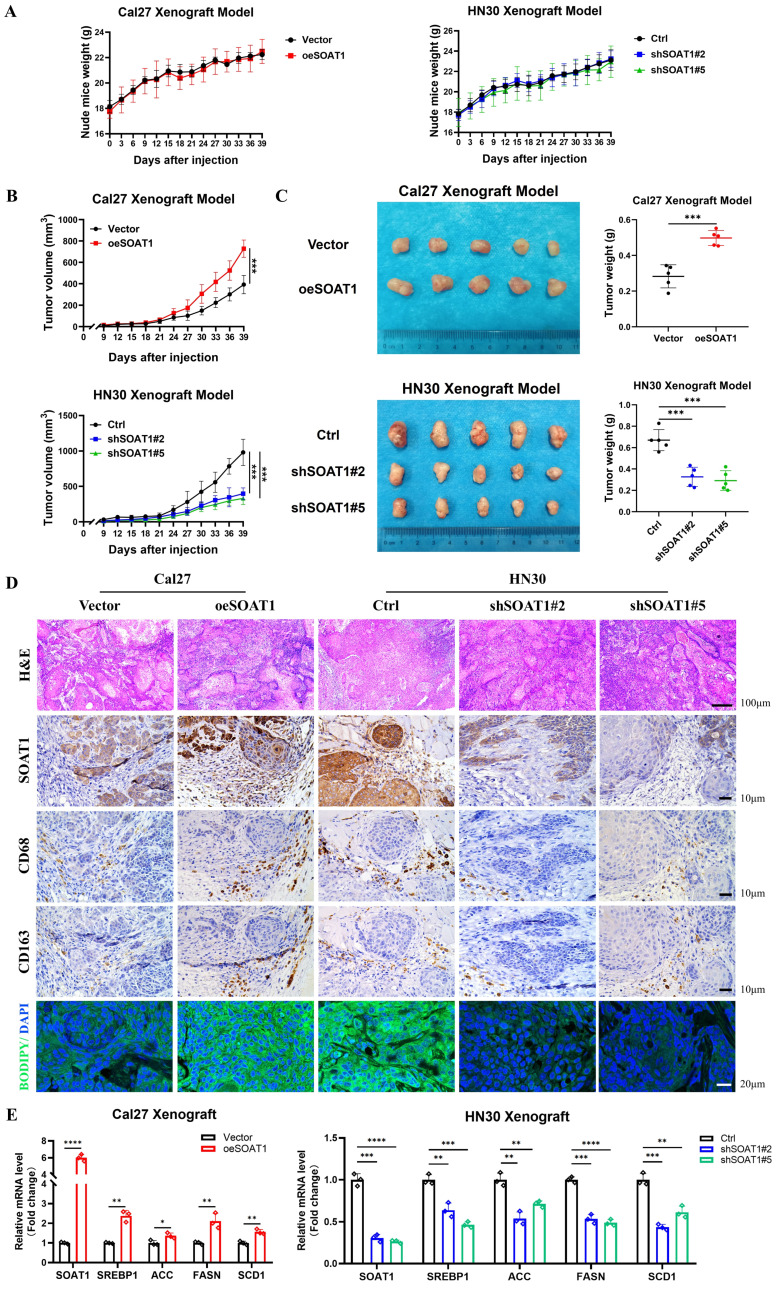
*SOAT1* augments tumor growth and facilitates M2 infiltration in xenograft. (A) Weight changes of nude mice after subcutaneous injections of Cal27-Vector/oeSOAT1 and HN30-Ctrl/shSOAT1#2/shSOAT1#5 cells in nude mice. (B) Growth curves of transplanted tumors after OSCC cells injection. (C) The transplanted tumors and their weights were obtained 39 days after OSCC cells subcutaneous injection. (D) Human tissues from OSCC patients were stained by H&E, immunohistochemistry (IHC) or BODIPY 493/503 (green)/DAPI (blue) via using *SOAT1*, *CD68* and *CD163* antibody. Scale bar, 100 μm for H&E, 10 μm for IHC staining and 20 μm for fluorescence imaging. (E) RT-qPCR analysis of lipid metabolism genes downstream of *SOAT1*. The asterisks represented the statistical *p*-value (*p < 0.05, **p < 0.01, ***p < 0.001, ****p < 0.0001).

For *in vivo* validation, xenografts were constructed by injecting Cal27 cells with *SOAT1* overexpression, HN30 cells with *SOAT1* knockdown, and corresponding control cells into the axillary subcutis of nude mice. We subsequently observed and recorded the body weight of the mice and the growth of the transplanted tumors. The treatment of nude mice is shown in **Figure S8**. The weight of the mice increased steadily after OSCC cell inoculation, and there was no significant difference between the groups, indicating that the mice grew normally **(Figure 7A)**. Cal27 cells with overexpression of *SOAT1* formed transplanted tumors subcutaneously in nude mice that were significantly larger in volume and weight than those in the control group, whereas the tumor volume in the HN30-shSOAT1 group was obviously smaller than that in the Ctrl group **(Figure 7B-C)**, suggesting that *SOAT1* exerts an essential oncogenic role *in vivo* and encourages the growth of OSCC transplanted tumors.

Tumors were embedded in paraffin and sectioned, and the expression of *SOAT1*, *CD68*, *CD163* and LDs in the tumors was detected by H&E, lipid droplet staining and IHC. The group with overexpressed *SOAT1* displayed a considerable increase in positive staining for *SOAT1*, while the knockdown group primarily showed negative *SOAT1* staining. Correspondingly, the expression of LDs and M2 TAMs infiltration was remarkably increased in the overexpression group, while greatly decreased in the knockdown group **(Figure 7D)**. Total RNA was extracted from xenograft tumor tissues to detect changes in mRNA levels of *SREBP1* and its downstream enzymes. In the group of overexpression *SOAT1*, the expression of *SREBP1*, *ACC*, *FASN* and *SCD1* was noticeably elevated, while it was dramatically suppressed by the down-regulation of *SOAT1* expression **(Figure 7E)**. It adequately demonstrated the pivotal regulatory role of *SOAT1* in lipid metabolism and M2 TAMs infiltration *in vivo*. Additionally, it was shown that *SOAT1* accelerated the progression of OSCC *in vivo* through the promotion of lipid metabolism and the increase of M2 TAMs infiltration.

## 4. Discussion

Oral malignancy is a significant disease that poses a substantial threat to human life and quality of life globally, characterized by high mortality and recurrence rates. Among the various types of oral cancer, OSCC stands out as the most prevalent 39. OSCC is a health challenge that has garnered considerable global attention, with an alarming number of over 350,000 new cases being diagnosed annually, leading to a staggering 170,000 deaths 10.

This particular ailment exerts significant strain on the societal framework. Given the comparatively low 5-year survival rate and elevated risk of recurrence, this unique type of cancer presents a great test for the medical community 40.

The development of OSCC is known to be significantly influenced by lipid metabolic reprogramming 10. *SOAT1*, located in the endoplasmic reticulum of cells and catalyzes the formation of cholesteryl esters 41, is up-regulated in various cancers 42. In studies of hepatocellular carcinoma, the knockdown of *SOAT1* was observed to alter the intracellular distribution pattern of cholesterol and exert an effective inhibitory effect on the growth and migration of hepatocellular carcinoma cells 43. *SREBP1* is a protein that plays a central role in lipid metabolism, specifically in mediating adipogenesis. In a normal physiological environment, the cholesterol in the endoplasmic reticulum (ER) membrane controls the activity of *SREBP* through a negative feedback loop 12. Inhibition of *SOAT1* reduces CE synthesis and LDs production, resulting in the accumulation of cholesterol in ER membranes. This, in turn, triggers the feedback inhibition of *SREBP* function. After conducting experimental research, we revealed that the expression level of *SOAT1* in OSCC positively regulates the expression of *SREBP1* and its downstream enzymes, *ACC*, *FASN*, and *SCD1*. The main end products of fatty acid synthesis, palmitate and oleic acid, which are controlled by *FASN* and *SCD1*, have been demonstrated to decrease cell death caused by *SOAT1* knockdown 12, which suggests that the growth inhibition caused by *SOAT1* knockdown is achieved by inhibiting *SREBP1* and its downstream enzymes. Taken together, we have clarified an important mechanism by which *SOAT1* promotes OSCC tumor growth. Specifically, the cholesterol esterification process mediated by *SOAT1* can reduce the inhibitory effect on *SREBP1* by decreasing cellular cholesterol levels, up-regulate the expression of downstream enzymes of *SREBP1*, such as *ACC*, *FASN*, and *SCD1*, promoting fatty acid synthesis and further advancing the progression of OSCC.

We observed a positive correlation between the expression levels of *SOAT1* and the infiltration of M2 TAMs in clinical specimens from patients with OSCC. These results indicate that *SOAT1* might be a driver of M2 TAMs infiltration in OSCC. Interestingly, this hypothesis was further supported by *in vivo* and *in vitro* experiments. It was shown that overexpression of *SOAT1* promotes M2-like polarization of TAMs and M2 TAMs infiltration of grafts. Subsequently, we conducted a preliminary investigation focusing on the mechanism by which *SOAT1* promotes TAMs M2-like polarization.

TAMs are a collection of multiple cell types that exhibit different functional roles under equilibrium and pathological conditions. This diversity is influenced by a variety of factors, including changes in metabolic processes and soluble molecules released by tumor cells 44. Recent studies have shown that lipid metabolism plays a key role in the differentiation and activation of TAMs 15. In particular, the process of cholesterol efflux has a significant impact on the phenotypic remodeling of TAMs and tumorigenesis 45. In this process, *ABCA1* and *ABCG1* are identified as key cholesterol efflux transporters 46. Research has shown that the reduction of cholesterol levels can promote the polarization of TAMs from M2 to M1 type, which involves the decrease in the expression level of *ABCA1* 33. In our study, we observed an increase in the mRNA levels of *ABCA1* and *ABCG1* in macrophages through conditioned medium cultivation. This finding suggests that the overexpression of *SOAT1* in OSCC may interfere with the cholesterol efflux of TAMs, thereby affecting their polarization state. In addition, TAMs can be activated and polarized by soluble molecules released by tumor cells, which in turn accelerates tumor progression and metastasis 44. Our analysis of four OSCC cell lines revealed a consistent trend of changes in CTSK mRNA expression with *SOAT1* expression. Using ELISA assay, we confirmed the role of *SOAT1* in promoting *CTSK* secretion. Flow cytometry results showed the involvement of CTSK in the effect of *SOAT1* on macrophage M2 polarization. In colorectal cancer models, it is known that tumor-secreted *CTSK* can bind to Toll-like receptor 4 (TLR4) on the surface of macrophages and promote M2 polarization through a mammalian target of rapamycin (mTOR)-dependent pathway 34. Therefore, we speculate that in OSCC, *SOAT1* enhances its interaction with *TLR4* on the surface of macrophages by increasing *CTSK* secretion, activating the mTOR pathway, and thus promoting M2 polarization. Based on the latest research progress and our experimental data, we propose two hypotheses to explain how *SOAT1* promotes the polarization of TAMs towards the M2 type. However, the specific molecular mechanisms behind these hypotheses still require further experimental verification.

In summary, this study provides compelling evidence supporting the pivotal oncogenic role of *SOAT1* in the progression of OSCC. We revealed that *SOAT1* was closely associated with a poor prognosis and M2 TAMs infiltration in OSCC. Through targeted interference with *SOAT1* expression levels in OSCC cells, we found that *SOAT1* promotes OSCC progression by increasing the proliferation, tumor sphere formation, migration, and invasive ability of OSCC cells. *SOAT1* facilitates OSCC progression by up-regulating the SREBP1-regulated adipogenic pathway and activating the PI3K/AKT/mTOR pathway. Apart from the above findings, we have successfully clarified the regulatory function of the transcription factor *ETS1* on *SOAT1*. Notably, this study has demonstrated the preliminary mechanism of *ETS1* in regulating *SOAT1* expression and participating in OSCC progression for the first time, providing a new molecular foundation for the treatment of OSCC. We have confirmed the critical oncogenic role of *SOAT1* in OSCC and identified it as a promising therapeutic target for OSCC. This work is anticipated to open up new avenues for metabolic and immunotherapeutic strategies for OSCC.

Our findings expand upon previous studies on the biology of *SOAT1*, but there are still some limitations to consider. Firstly, as *SOAT1* plays a significant role in driving the progression of OSCC, targeting *SOAT1* has the potential to be an effective treatment for OSCC. Further research is needed on potential inhibitors of *SOAT1*. Secondly, this study primarily investigated the induced polarization effect of *SOAT1* on TAMs in OSCC, without delving into the specific mechanisms of promoting M2-like polarization of TAMs. Based on the results of the *in vitro* experiments, it is evident that *CTSK* only partially reversed the pro-M2 polarization effect of *SOAT1*, and it is also clear that there are other mechanisms besides *CTSK* that contribute to the pro-M2 polarization of *SOAT1*. These will be the main areas of focus for our future research.

## 5. Conclusion

Based on the findings, we propose that *SOAT1* is regulated by *ETS1* and plays a crucial role in the development of OSCC by promoting lipid metabolism, PI3K/AKT/mTOR pathway and M2-like polarization of TAMs. We suggest that *SOAT1* is a promising target for OSCC therapy with tremendous potential.

## Supplementary Material

Supplementary figures.

## Figures and Tables

**Table 1:** Sequences for shRNA vectors

shRNA oligos	Sequences
shSOAT1#1	5'-CCACGTCATACTCCAACTATT-3'
shSOAT1#2	5'-GAACGTGCCTCGGGTACTAAA-3'
shSOAT1#5	5'-TGGTCCATGACTGGCTATATT-3'
shETS1#1	5'-GTGCAGATGTCCCACTATTAA-3'
shETS1#4	5'-GACCGTGCTGACCTCAATAAG-3'
shETS1#5	5'-ATCCCGCTATACCTCGGATTA-3'

**Table 2:** Primers for qPCR

Primers	Forward	Reverse
*SOAT1*	5'-GCTCGTGTTCTGGTCCTATGTG-3'	5'-TAGAACATCCTGTCACCAAAGCG-3'
*SREBP1*	5'-ACTTCTGGAGGCATCGCAAGCA-3'	5'-AGGTTCCAGAGGAGGCTACAAG-3'
*ACC*	5'-TTCACTCCACCTTGTCAGCGGA-3'	5'-GTCAGAGAAGCAGCCCATCACT-3'
*FASN*	5'-TTCTACGGCTCCACGCTCTTCC-3'	5'-GAAGAGTCTTCGTCAGCCAGGA-3'
*SCD1*	5'-CCTGGTTTCACTTGGAGCTGTG-3'	5'-TGTGGTGAAGTTGATGTGCCAGC-3'
*CD68*	5'-CGAGCATCATTCTTTCACCAGCT-3'	5'-ATGAGAGGCAGCAAGATGGACC-3'
*CD86*	5'-CCATCAGCTTGTCTGTTTCATTCC-3'	5'-GCTGTAATCCAAGGAATGTGGTC-3'
*IL-6*	5'- AGACAGCCACTCACCTCTTCAG -3'	5'- TTCTGCCAGTGCCTCTTTGCTG -3'
*CD206*	5'-AGCCAACACCAGCTCCTCAAGA-3'	5'-CAAAACGCTCGCGCATTGTCCA-3'
*IL-10*	5'-GACTTTAAGGGTTACCTGGGTTG-3'	5'-TCACATGCGCCTTGATGTCTG-3'
*ETS1*	5'-GAGTCAACCCAGCCTATCCAGA-3'	5'-GAGCGTCTGATAGGACTCTGTG-3'
*β-actin*	5′-CACCATTGGCAATGAGCGGTTC-3′	5′-AGGTCTTTGCGGATGTCCACGT-3′

**Table 3:** Primers for ChIP

Site	Forward	Reverse
Site 1	5'- GGTGGAAGTGTCAAGCAAGAT -3'	5'- CTGAGCATCCTATTGGTGGTC-3'
Site 2	5′- GTGAGAAGCTTCCTTGGCAG-3′	5′- CGTCGCCTGCTAAGCTAAAG-3′

## Abbreviations

OSCC: oral squamous cell carcinoma
TAMs: tumor associated macrophages
SOAT1: sterol O-acyltransferase 1
SREBP1: sterol regulatory element-binding protein-1
ETS1: ETS proto-oncogene 1
HNSCC: head and neck squamous cell carcinoma
CE: cholesteryl esters
LDs: lipid droplets
TME: the tumor microenvironment
TCGA: The Cancer Genome Atlas
KM: Kaplan-Meier
FBS: fetal bovine serum
PMA: phorbol 12-myristate 13-acetate
cDNA: complementary DNA
PBS: phosphate-buffered saline
IHC: immunohistochemistry
RT-qPCR: Real-Time Quantitative PCR
CCK-8: Cell Counting Kit-8
OD: optical density
CM: conditioned medium
CTSK: cathepsin K
ChIP: chromatin immunoprecipitation
DLR: promoter dual-luciferase reporter assay
RLU: relative light units
ACC: acetyl-CoA carboxylase
FASN: fatty acid synthase
SCD1: stearoyl-CoA desaturase-1
TLR4: toll-like receptor 4
mTOR: mammalian target of rapamycin
KEGG: Kyoto Encyclopedia of Genes and Genomes

## References

[1] Gormley M, Dudding T, Sanderson E, Martin RM, Thomas S, Tyrrell J (2020). A multivariable Mendelian randomization analysis investigating smoking and alcohol consumption in oral and oropharyngeal cancer. Nat Commun.

[2] Zou C, Lv X, Wei H, Wu S, Song J, Tang Z (2022). Long non-coding RNA LINC00472 inhibits oral squamous cell carcinoma via miR-4311/GNG7 axis. Bioengineered.

[3] Hu S, Lu H, Xie W, Wang D, Shan Z, Xing X (2022). TDO2+ myofibroblasts mediate immune suppression in malignant transformation of squamous cell carcinoma. J Clin Invest.

[4] Tang Q, Xie M, Yu S, Zhou X, Xie Y, Chen G (2019). Periodic Oxaliplatin Administration in Synergy with PER2-Mediated PCNA Transcription Repression Promotes Chronochemotherapeutic Efficacy of OSCC. Adv Sci (Weinh).

[5] Fan T, Wang X, Zhang S, Deng P, Jiang Y, Liang Y (2022). NUPR1 promotes the proliferation and metastasis of oral squamous cell carcinoma cells by activating TFE3-dependent autophagy. Signal transduction and targeted therapy.

[6] Morand GB, Broglie MA, Schumann P, Huellner MW, Rupp NJ (2020). Histometabolic Tumor Imaging of Hypoxia in Oral Cancer: Clinicopathological Correlation for Prediction of an Aggressive Phenotype. Front Oncol.

[7] da Silva SD, Marchi FA, Su J, Yang L, Valverde L, Hier J (2021). Co-Overexpression of TWIST1-CSF1 Is a Common Event in Metastatic Oral Cancer and Drives Biologically Aggressive Phenotype. Cancers (Basel).

[8] Tsai YT, Chen WC, Hsu CM, Tsai MS, Chang GH, Lee YC (2021). Survival-Weighted Health Profiles in Patients Treated for Advanced Oral Cavity Squamous Cell Carcinoma. Front Oncol.

[9] Guo X, Zhou S, Yang Z, Li ZA, Hu W, Dai L (2022). Comprehensive Analysis of Sterol O-Acyltransferase 1 as a Prognostic Biomarker and Its Association With Immune Infiltration in Glioma. Front Oncol.

[10] Chen X, Liu Q, Chen Y, Wang L, Yang R, Zhang W (2022). Carboxylesterase 2 induces mitochondrial dysfunction via disrupting lipid homeostasis in oral squamous cell carcinoma. Mol Metab.

[11] Oni TE, Biffi G, Baker LA, Hao Y, Tonelli C, Somerville TDD (2020). SOAT1 promotes mevalonate pathway dependency in pancreatic cancer. J Exp Med.

[12] Geng F, Cheng X, Wu X, Yoo JY, Cheng C, Guo JY (2016). Inhibition of SOAT1 Suppresses Glioblastoma Growth via Blocking SREBP-1-Mediated Lipogenesis. Clinical cancer research: an official journal of the American Association for Cancer Research.

[13] Sbiera S, Leich E, Liebisch G, Sbiera I, Schirbel A, Wiemer L (2015). Mitotane Inhibits Sterol-O-Acyl Transferase 1 Triggering Lipid-Mediated Endoplasmic Reticulum Stress and Apoptosis in Adrenocortical Carcinoma Cells. Endocrinology.

[14] Wang Y, Xu J, Fang Y, Gu J, Zhao F, Tang Y (2022). Comprehensive analysis of a novel signature incorporating lipid metabolism and immune-related genes for assessing prognosis and immune landscape in lung adenocarcinoma. Front Immunol.

[15] Su P, Wang Q, Bi E, Ma X, Liu L, Yang M (2020). Enhanced Lipid Accumulation and Metabolism Are Required for the Differentiation and Activation of Tumor-Associated Macrophages. Cancer Res.

[16] Neubert NJ, Schmittnaegel M, Bordry N, Nassiri S, Wald N, Martignier C (2018). T cell-induced CSF1 promotes melanoma resistance to PD1 blockade. Sci Transl Med.

[17] Zhang Q, Huang F, Yao Y, Wang J, Wei J, Wu Q (2019). Interaction of transforming growth factor-beta-Smads/microRNA-362-3p/CD82 mediated by M2 macrophages promotes the process of epithelial-mesenchymal transition in hepatocellular carcinoma cells. Cancer Sci.

[18] Mantovani A, Marchesi F, Malesci A, Laghi L, Allavena P (2017). Tumour-associated macrophages as treatment targets in oncology. Nat Rev Clin Oncol.

[19] Wei Z, Zhang X, Yong T, Bie N, Zhan G, Li X (2021). Boosting anti-PD-1 therapy with metformin-loaded macrophage-derived microparticles. Nat Commun.

[20] Zhang J, Li H, Wu Q, Chen Y, Deng Y, Yang Z (2019). Tumoral NOX4 recruits M2 tumor-associated macrophages via ROS/PI3K signaling-dependent various cytokine production to promote NSCLC growth. Redox Biol.

[21] Zeng J, Ye Z, Shi S, Liang Y, Meng Q, Zhang Q (2023). Targeted inhibition of eIF5A(hpu) suppresses tumor growth and polarization of M2-like tumor-associated macrophages in oral cancer. Cell death & disease.

[22] Dittmer J (2015). The role of the transcription factor Ets1 in carcinoma. Semin Cancer Biol.

[23] Vishnoi K, Viswakarma N, Rana A, Rana B (2020). Transcription Factors in Cancer Development and Therapy. Cancers (Basel).

[24] Sakamoto K, Endo K, Sakamoto K, Kayamori K, Ehata S, Ichikawa J (2021). EHF suppresses cancer progression by inhibiting ETS1-mediated ZEB expression. Oncogenesis.

[25] Kim CJ, Lee CG, Jung JY, Ghosh A, Hasan SN, Hwang SM (2018). The Transcription Factor Ets1 Suppresses T Follicular Helper Type 2 Cell Differentiation to Halt the Onset of Systemic Lupus Erythematosus. Immunity.

[26] Li H, Zeng C, Shu C, Cao Y, Shao W, Zhang M (2022). Laminins in tumor-derived exosomes upregulated by ETS1 reprogram omental macrophages to promote omental metastasis of ovarian cancer. Cell death & disease.

[27] Newman AM, Steen CB, Liu CL, Gentles AJ, Chaudhuri AA, Scherer F (2019). Determining cell type abundance and expression from bulk tissues with digital cytometry. Nat Biotechnol.

[28] Liang L, Liu Y, Jiang S, Huang J, He H, Shen L (2022). Novel Circulating Tumour Cell-Related Risk Model Indicates Prognosis and Immune Infiltration in Lung Adenocarcinoma. J Immunol Res.

[29] Jiang Y, Mao C, Yang R, Yan B, Shi Y, Liu X (2017). EGLN1/c-Myc Induced Lymphoid-Specific Helicase Inhibits Ferroptosis through Lipid Metabolic Gene Expression Changes. Theranostics.

[30] Tewari D, Patni P, Bishayee A, Sah AN, Bishayee A (2022). Natural products targeting the PI3K-Akt-mTOR signaling pathway in cancer: A novel therapeutic strategy. Semin Cancer Biol.

[31] Wang J, Jiang C, Li N, Wang F, Xu Y, Shen Z (2020). The circEPSTI1/mir-942-5p/LTBP2 axis regulates the progression of OSCC in the background of OSF via EMT and the PI3K/Akt/mTOR pathway. Cell Death Dis.

[32] Harsha C, Banik K, Ang HL, Girisa S, Vikkurthi R, Parama D (2020). Targeting AKT/mTOR in Oral Cancer: Mechanisms and Advances in Clinical Trials. Int J Mol Sci.

[33] Jin H, He Y, Zhao P, Hu Y, Tao J, Chen J (2019). Targeting lipid metabolism to overcome EMT-associated drug resistance via integrin beta3/FAK pathway and tumor-associated macrophage repolarization using legumain-activatable delivery. Theranostics.

[34] Li R, Zhou R, Wang H, Li W, Pan M, Yao X (2019). Gut microbiota-stimulated cathepsin K secretion mediates TLR4-dependent M2 macrophage polarization and promotes tumor metastasis in colorectal cancer. Cell Death Differ.

[35] Woo Y, Lee HJ, Kim J, Kang SG, Moon S, Han JA (2021). Rapamycin Promotes ROS-Mediated Cell Death via Functional Inhibition of xCT Expression in Melanoma Under gamma-Irradiation. Front Oncol.

[36] Teng JF, Mei QB, Zhou XG, Tang Y, Xiong R, Qiu WQ (2020). Polyphyllin VI Induces Caspase-1-Mediated Pyroptosis via the Induction of ROS/NF-kappaB/NLRP3/GSDMD Signal Axis in Non-Small Cell Lung Cancer. Cancers (Basel).

[37] Wang S, Fu JL, Hao HF, Jiao YN, Li PP, Han SY (2021). Metabolic reprogramming by traditional Chinese medicine and its role in effective cancer therapy. Pharmacol Res.

[38] Zhang L, Xiong YL, Ren YL, Liu XW, Si Y, Liu Y (2022). [Mechanism of polyphyllin A in inhibition of proliferation and induction of apoptosis of gastric cancer cells by directly targeting ETS-1]. Zhongguo Zhong Yao Za Zhi.

[39] Gohara S, Shinohara K, Yoshida R, Kariya R, Tazawa H, Hashimoto M (2022). An oncolytic virus as a promising candidate for the treatment of radioresistant oral squamous cell carcinoma. Mol Ther Oncolytics.

[40] Huang GZ, Wu QQ, Zheng ZN, Shao TR, Chen YC, Zeng WS (2020). M6A-related bioinformatics analysis reveals that HNRNPC facilitates progression of OSCC via EMT. Aging (Albany NY).

[41] Zhu T, Wang Z, Zou T, Xu L, Zhang S, Chen Y (2021). SOAT1 Promotes Gastric Cancer Lymph Node Metastasis Through Lipid Synthesis. Front Pharmacol.

[42] Gu L, Zhu Y, Lin X, Tan X, Lu B, Li Y (2020). Stabilization of FASN by ACAT1-mediated GNPAT acetylation promotes lipid metabolism and hepatocarcinogenesis. Oncogene.

[43] Jiang Y, Sun A, Zhao Y, Ying W, Sun H, Yang X (2019). Proteomics identifies new therapeutic targets of early-stage hepatocellular carcinoma. Nature.

[44] Pan Y, Yu Y, Wang X, Zhang T (2020). Tumor-Associated Macrophages in Tumor Immunity. Front Immunol.

[45] Goossens P, Rodriguez-Vita J, Etzerodt A, Masse M, Rastoin O, Gouirand V (2019). Membrane Cholesterol Efflux Drives Tumor-Associated Macrophage Reprogramming and Tumor Progression. Cell Metab.

[46] Xu Y, Xu Y, Zhu Y, Sun H, Juguilon C, Li F (2020). Macrophage miR-34a Is a Key Regulator of Cholesterol Efflux and Atherosclerosis. Mol Ther.

