# Supplementary material for: ETS1-mediated Regulation of SOAT1 Enhances the Malignant Phenotype of Oral Squamous Cell Carcinoma and Induces Tumor-associated Macrophages M2-like Polarization
Source: Int J Biol Sci. 2024 Jun 11;20(9):3372–92. doi: 10.7150/ijbs.93815 (PMC11234219; doi:10.7150/ijbs.93815)
Supplement: Supplementary file 1 — Supplementary figures. [file ijbsv20p3372s1.pdf]

FIGURE S1

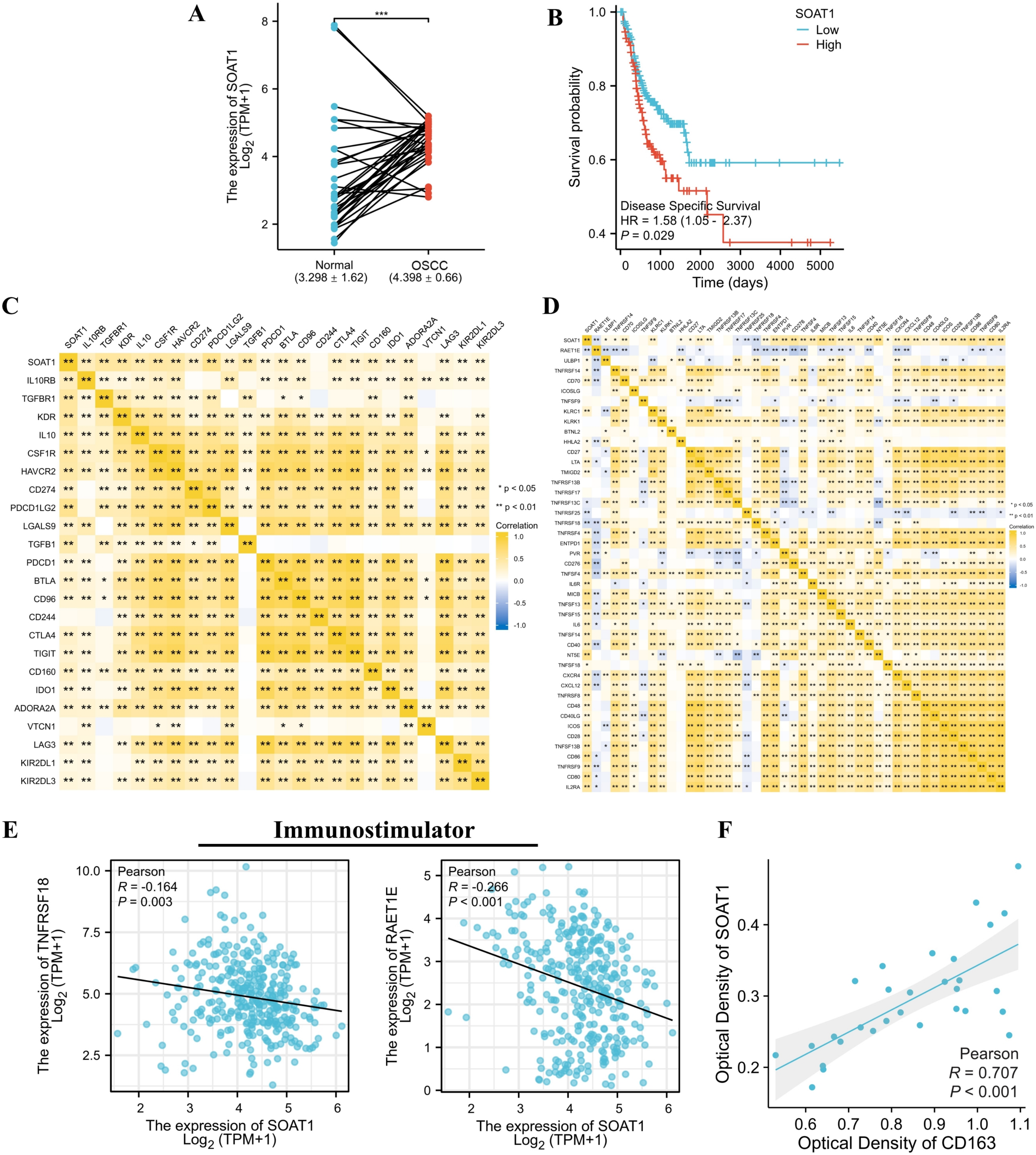
A
***
The expression of SOAT1
Log2 (TPM+1)
Normal
(3.298 ± 1.62)
OSCC
(4.398 ± 0.66)
B
SOAT1
Low
High
Survival probability
Disease Specific Survival
HR = 1.58 (1.05 - 2.37)
P = 0.029
Time (days)
C
SOAT1 IL10RB TGFBR1 KDR IL10 CSF1R HAVCR2 CD274 PDCD1LG2 LGALS9 TGFB1 PDCD1 BTLA CD96 CD244 CTLA4 TIGIT CD160 IDO1 ADORA2A VTCN1 LAG3 KIR2DL1 KIR2DL3
* p < 0.05
** p < 0.01
Correlation
D
SOAT1 RAET1E ULBP1 TNFRSF14 CD70 ICOSLG TNFSF9 KLRC1 KLRK1 BTNL2 HHLA2 CD27 LTA TMIGD2 TNFRSF13B TNFRSF17 TNFRSF13C TNFRSF25 TNFRSF18 TNFRSF4 ENTPD1 PVR CD276 TNFSF4 IL6R MICB TNFSF13 TNFSF15 IL6 TNFSF14 CD40 NT5E TNFSF18 CXCR4 CXCL12 TNFRSF8 CD48 CD40LG ICOS CD28 TNFSF13B CD86 TNFRSF9 CD80 IL2RA
E
Immunostimulator
Pearson
R = -0.164
P = 0.003
The expression of TNFRSF18
Log2 (TPM+1)
The expression of SOAT1
Log2 (TPM+1)
Pearson
R = -0.266
P < 0.001
The expression of RAET1E
Log2 (TPM+1)
The expression of SOAT1
Log2 (TPM+1)
F
Optical Density of SOAT1
Pearson
R = 0.707
P < 0.001
Optical Density of CD163

FIGURE S2

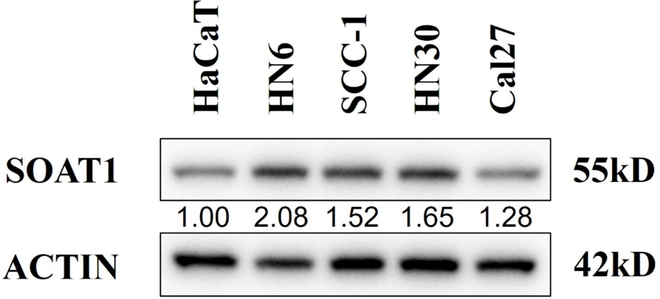
HaCaT
HN6
SCC-1
HN30
Cal27
SOAT1
55kD
1.00
2.08
1.52
1.65
1.28
ACTIN
42kD

FIGURE S3

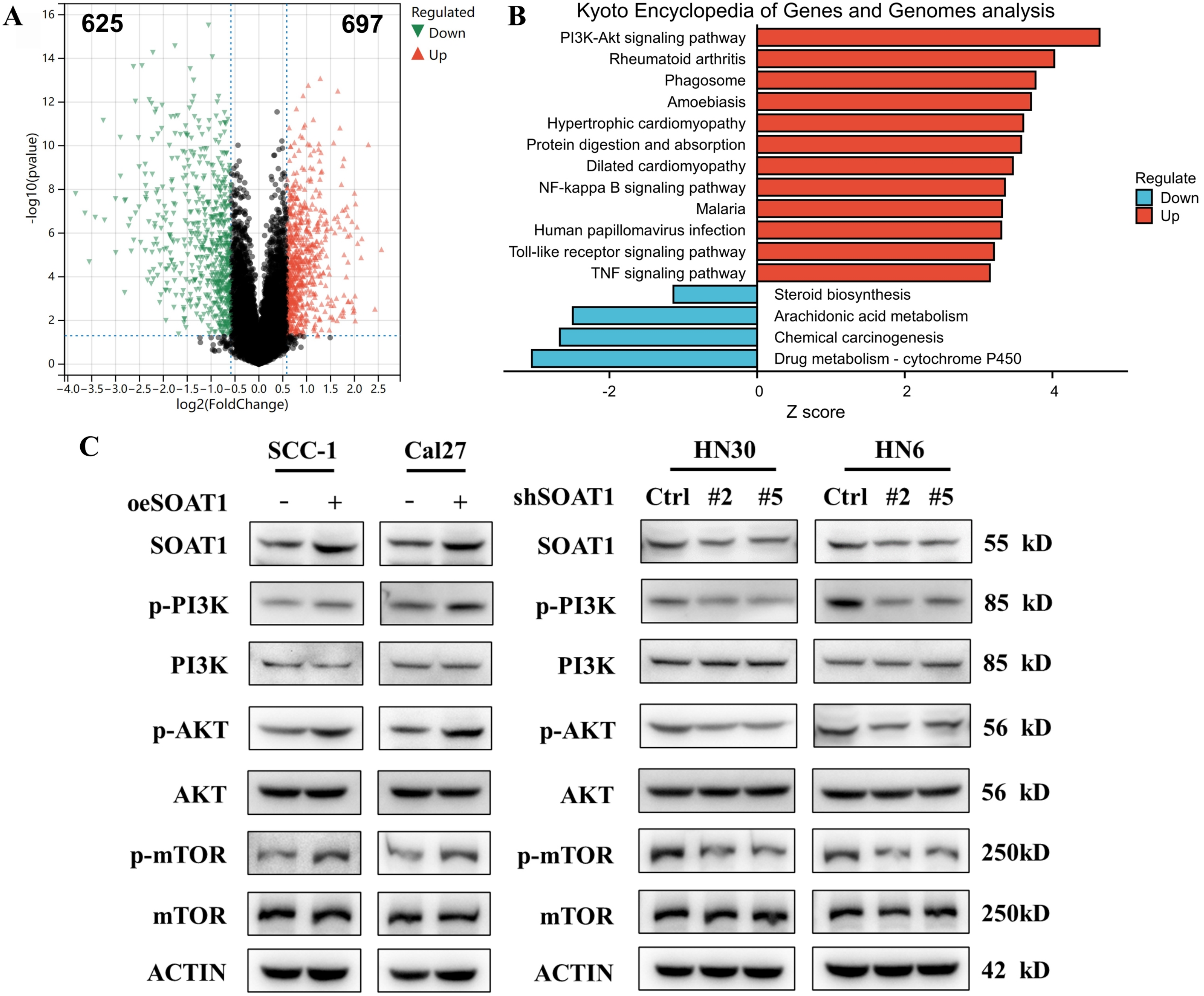
A
625
697
Regulated
Down
Up
-log10(pvalue)
log2(FoldChange)
B
Kyoto Encyclopedia of Genes and Genomes analysis
PI3K-Akt signaling pathway
Rheumatoid arthritis
Phagosome
Amoebiasis
Hypertrophic cardiomyopathy
Protein digestion and absorption
Dilated cardiomyopathy
NF-kappa B signaling pathway
Malaria
Human papillomavirus infection
Toll-like receptor signaling pathway
TNF signaling pathway
Steroid biosynthesis
Arachidonic acid metabolism
Chemical carcinogenesis
Drug metabolism - cytochrome P450
Regulate
Down
Up
Z score
C
SCC-1
Cal27
HN30
HN6
oeSOAT1
shSOAT1
Ctrl #2 #5
SOAT1
p-PI3K
PI3K
p-AKT
AKT
p-mTOR
mTOR
ACTIN
55 kD
85 kD
85 kD
56 kD
56 kD
250kD
250kD
42 kD

FIGURE S4

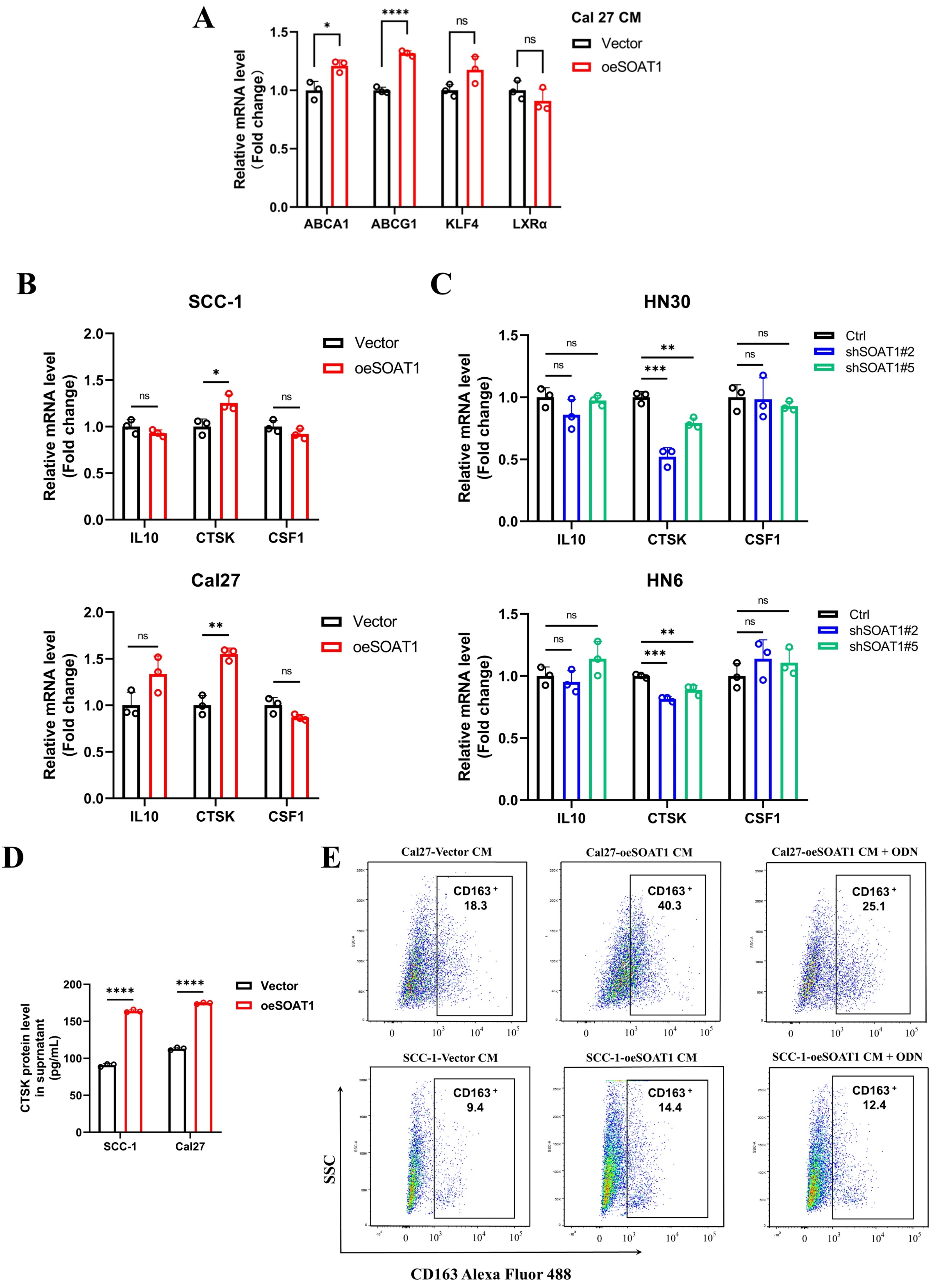

A
Cal 27 CM
Vector
oeSOAT1
Relative mRNA level (Fold change)
ABCA1
ABCG1
KLF4
LXRα
ns
B
SCC-1
Cal27
Relative mRNA level (Fold change)
IL10
CTSK
CSF1
Vector
oeSOAT1
C
HN30
HN6
Ctrl
shSOAT1#2
shSOAT1#5
D
CTSK protein level in suprnatant (pg/mL)
SCC-1
Cal27
E
Cal27-Vector CM
Cal27-oeSOAT1 CM
Cal27-oeSOAT1 CM + ODN
CD163 + 18.3
CD163 + 40.3
CD163 + 25.1
SCC-1-Vector CM
SCC-1-oeSOAT1 CM
SCC-1-oeSOAT1 CM + ODN
CD163 + 9.4
CD163 + 14.4
CD163 + 12.4
SSC
CD163 Alexa Fluor 488

FIGURE S5

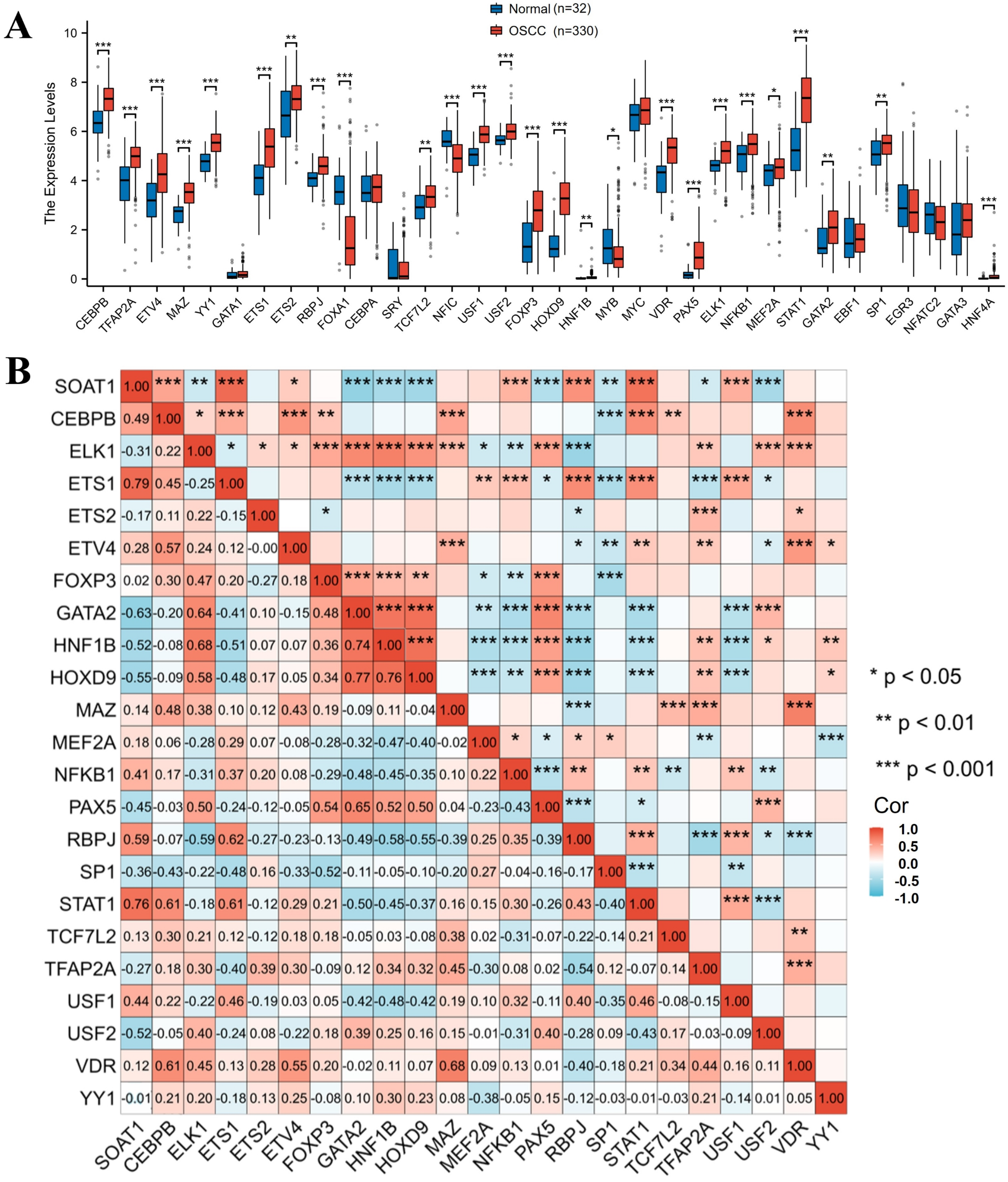
A
Normal (n=32)
OSCC (n=330)
The Expression Levels
0
2
4
6
8
10
CEBPB TFAP2A ETV4 MAZ YY1 GATA1 ETS1 ETS2 RBPJ FOXA1 CEBPA SRY TCF7L2 NFIC USF1 USF2 FOXP3 HOXD9 HNF1B MYB MYC VDR PAX5 ELK1 NFKB1 MEF2A STAT1 GATA2 EBF1 SP1 EGR3 NFATC2 GATA3 HNF4A
B
SOAT1 1.00
CEBPB 0.49 1.00
ELK1 -0.31 0.22 1.00
ETS1 0.79 0.45 -0.25 1.00
ETS2 -0.17 0.11 0.22 -0.15 1.00
ETV4 0.28 0.57 0.24 0.12 -0.00 1.00
FOXP3 0.02 0.30 0.47 0.20 -0.27 0.18 1.00
GATA2 -0.63 -0.20 0.64 -0.41 0.10 -0.15 0.48 1.00
HNF1B -0.52 -0.08 0.68 -0.51 0.07 0.07 0.36 0.74 1.00
HOXD9 -0.55 -0.09 0.58 -0.48 0.17 0.05 0.34 0.77 0.76 1.00
MAZ 0.14 0.48 0.38 0.10 0.12 0.43 0.19 -0.09 0.11 -0.04 1.00
MEF2A 0.18 0.06 -0.28 0.29 0.07 -0.08 -0.28 -0.32 -0.47 -0.40 -0.02 1.00
NFKB1 0.41 0.17 -0.31 0.37 0.20 0.08 -0.29 -0.48 -0.45 -0.35 0.10 0.22 1.00
PAX5 -0.45 -0.03 0.50 -0.24 -0.12 -0.05 0.54 0.65 0.52 0.50 0.04 -0.23 -0.43 1.00
RBPJ 0.59 -0.07 -0.59 0.62 -0.27 -0.23 -0.13 -0.49 -0.58 -0.55 -0.39 0.25 0.35 -0.39 1.00
SP1 -0.36 -0.43 -0.22 -0.48 0.16 -0.33 -0.52 -0.11 -0.05 -0.10 -0.20 0.27 -0.04 -0.16 -0.17 1.00
STAT1 0.76 0.61 -0.18 0.61 -0.12 0.29 0.21 -0.50 -0.45 -0.37 0.16 0.15 0.30 -0.26 0.43 -0.40 1.00
TCF7L2 0.13 0.30 0.21 0.12 -0.12 0.18 0.18 -0.05 0.03 -0.08 0.38 0.02 -0.31 -0.07 -0.22 -0.14 0.21 1.00
TFAP2A -0.27 0.18 0.30 -0.40 0.39 0.30 -0.09 0.12 0.34 0.32 0.45 -0.30 0.08 0.02 -0.54 0.12 -0.07 0.14 1.00
USF1 0.44 0.22 -0.22 0.46 -0.19 0.03 0.05 -0.42 -0.48 -0.42 0.19 0.10 0.32 -0.11 0.40 -0.35 0.46 -0.08 -0.15 1.00
USF2 -0.52 -0.05 0.40 -0.24 0.08 -0.22 0.18 0.39 0.25 0.16 0.15 -0.01 -0.31 0.40 -0.28 0.09 -0.43 0.17 -0.03 -0.09 1.00
VDR 0.12 0.61 0.45 0.13 0.28 0.55 0.20 -0.02 0.11 0.07 0.68 0.09 0.13 0.01 -0.40 -0.18 0.21 0.34 0.44 0.16 0.11 1.00
YY1 -0.01 0.21 0.20 -0.18 0.13 0.25 -0.08 0.10 0.30 0.23 0.08 -0.38 -0.05 0.15 -0.12 -0.03 -0.01 -0.03 0.21 -0.14 0.01 0.05 1.00
SOAT1 CEBPB ELK1 ETS1 ETS2 ETV4 FOXP3 GATA2 HNF1B HOXD9 MAZ MEF2A NFKB1 PAX5 RBPJ SP1 STAT1 TCF7L2 TFAP2A USF1 USF2 VDR YY1
* p < 0.05
** p < 0.01
*** p < 0.001
Cor
1.0
0.5
0.0
-0.5
-1.0

FIGURE S6

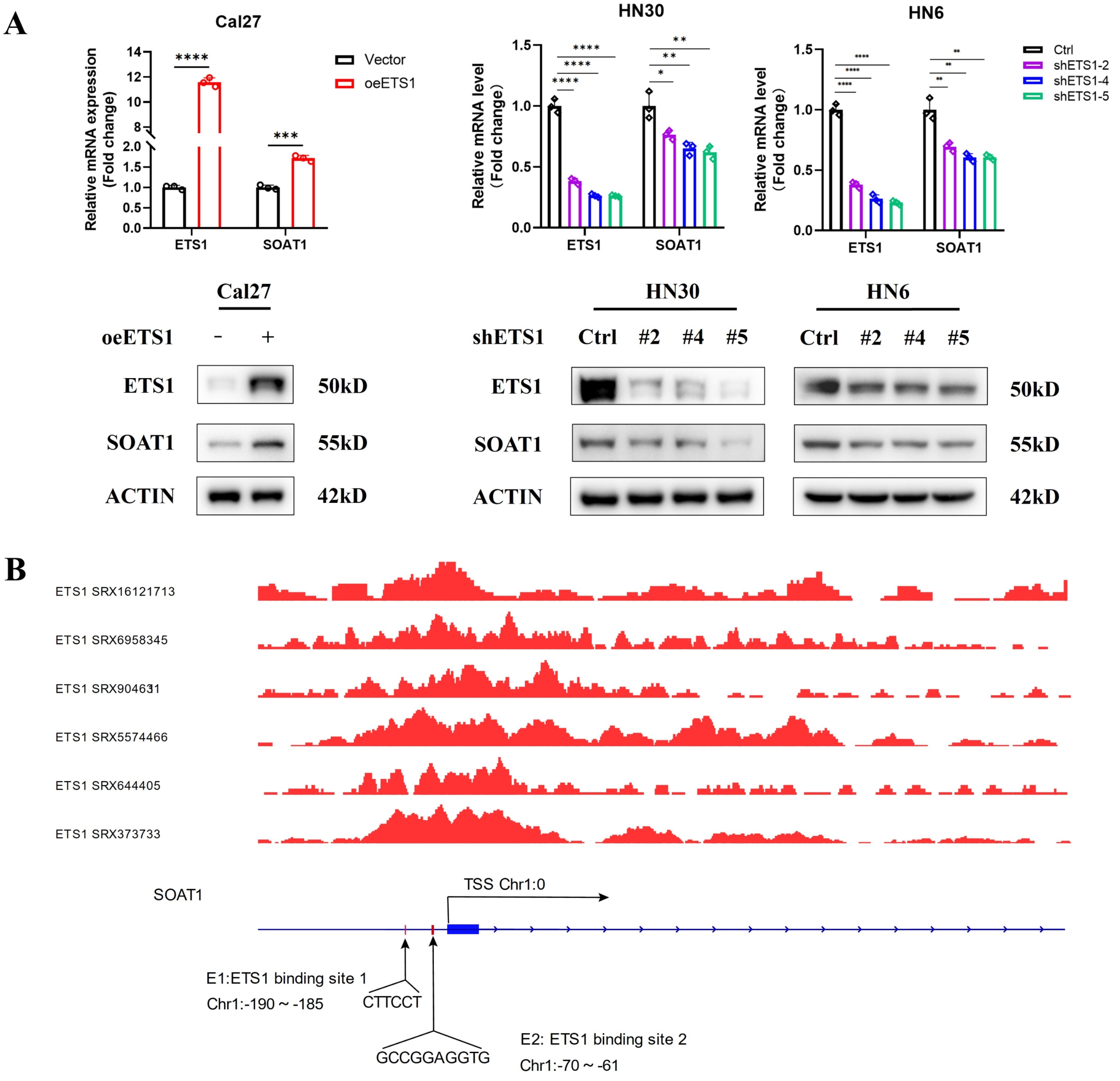
A
Cal27
Relative mRNA expression (Fold change)
Vector
oeETS1
****
***
ETS1
SOAT1
HN30
HN6
Relative mRNA level (Fold change)
Ctrl
shETS1-2
shETS1-4
shETS1-5
oeETS1
-
+
ETS1
SOAT1
ACTIN
50kD
55kD
42kD
shETS1
Ctrl
#2
#4
#5
B
ETS1 SRX16121713
ETS1 SRX6958345
ETS1 SRX904631
ETS1 SRX5574466
ETS1 SRX644405
ETS1 SRX373733
SOAT1
TSS Chr1:0
E1:ETS1 binding site 1
Chr1:-190 ~ -185
CTTCCT
GCCGGAGGTG
E2: ETS1 binding site 2
Chr1:-70 ~ -61

FIGURE S7

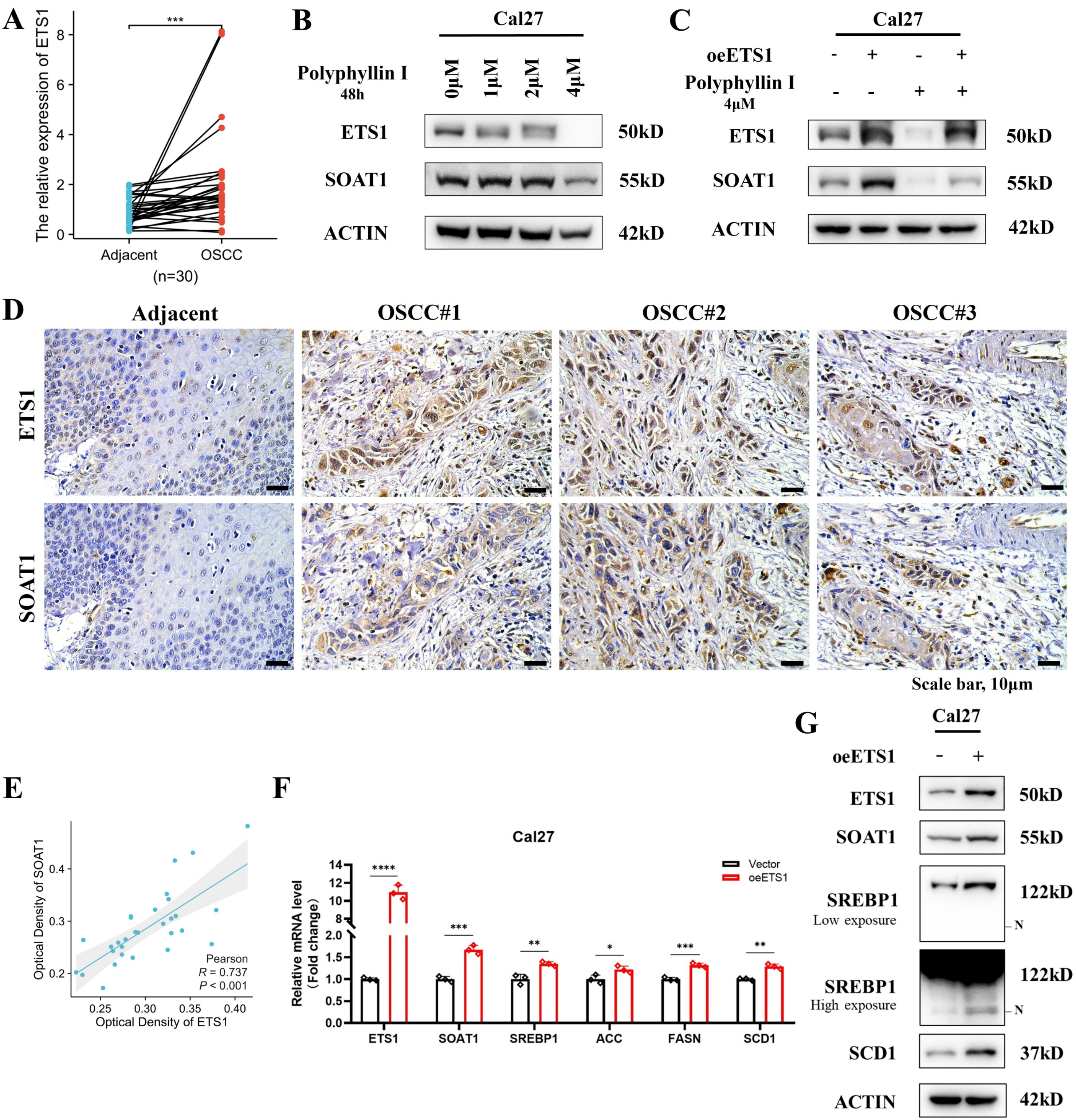
A
The relative expression of ETS1
***
Adjacent
OSCC
(n=30)
B
Cal27
Polyphyllin I
48h
0μM
1μM
2μM
4μM
ETS1
50kD
SOAT1
55kD
ACTIN
42kD
C
Cal27
oeETS1
Polyphyllin I
4μM
ETS1
50kD
SOAT1
55kD
ACTIN
42kD
D
Adjacent
OSCC#1
OSCC#2
OSCC#3
ETS1
SOAT1
Scale bar, 10μm
E
Optical Density of SOAT1
Optical Density of ETS1
Pearson
R = 0.737
P < 0.001
F
Cal27
Relative mRNA level (Fold change)
Vector
oeETS1
ETS1
SOAT1
SREBP1
ACC
FASN
SCD1
G
Cal27
oeETS1
ETS1
50kD
SOAT1
55kD
SREBP1
Low exposure
122kD
N
SREBP1
High exposure
122kD
N
SCD1
37kD
ACTIN
42kD

FIGURE S8

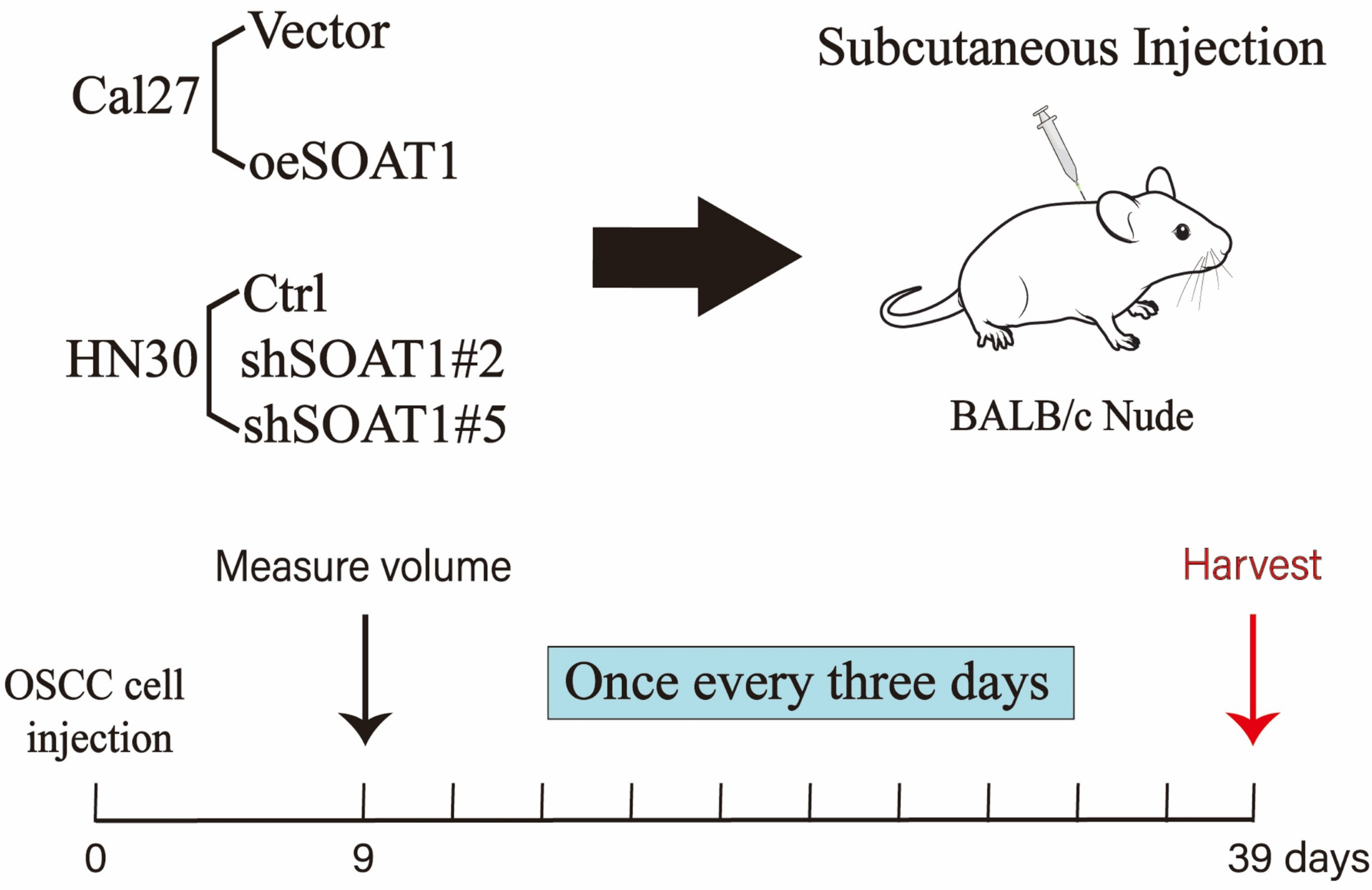
Vector
Cal27
oeSOAT1
Ctrl
HN30
shSOAT1#2
shSOAT1#5
Subcutaneous Injection
BALB/c Nude
Measure volume
Harvest
OSCC cell injection
Once every three days
0
9
39 days

Supplementary figure legends

Figure S1 (A) The expression of *SOAT1* in OSCC and normal paired samples. (B) Kaplan-Meier curves of disease-specific survival for high and low groups of *SOAT1* expression. (C) Heat map of *SOAT1* correlation with suppressive immunomodulatory genes. (D) Heat map of *SOAT1* correlation with stimulatory immunomodulatory genes. (E) Scatter plots of *SOAT1* correlation with stimulatory immunomodulatory genes. (F) IHC staining intensity correlation analysis of *SOAT1* and *CD163* in 30 pairs of clinical samples of OSCC paired with adjacent tissue. The asterisks represented the statistical p-value (*$p < 0.05$, **$p < 0.01$, ***$p < 0.001$).

Figure S2 Western Blot assay was performed to detect the protein expression level of SOAT1 in four OSCC cell lines and HaCaT cell.

Figure S3 (A) Volcano plot of differentially expressed genes in high and low groups of *SOAT1* expression in TCGA-OSCC. (B) KEGG analysis of DEGs between high and low expression groups. (C) The effects of overexpression and knockdown of *SOAT1* on the expression of p-PI3K, PI3K, p-AKT, AKT, p-mTOR and mTOR in OSCC cells were analyzed by Western Blot.

Figure S4 (A) RT-qPCR analysis of macrophages after cultured with CM of *SOAT1*-overexpressing Cal27 cell. (B) RT-qPCR analysis of pro-M2 polarization genes in *SOAT1* overexpressing OSCC cells. (C) RT-qPCR analysis of pro-M2 polarization genes in *SOAT1* knockdown OSCC cells. (D) ELISA assay to detect CTSK levels in the supernatants of SCC-1 and Cal27 cells overexpressing *SOAT1*. (E) Flow cytometry was used to assess the expression level of CD163 in macrophages after culture in conditioned medium and treatment with ODN. (ODN: Odanacatib, a CTSK-specific inhibitor) The asterisks represented the statistical p-value (ns = no significant; *$p < 0.05$, **$p < 0.01$, ***$p < 0.001$, ****$p < 0.0001$).

Figure S5 (A) Box plot display the expression levels of 34 potential upstream

transcription factors in OSCC (n=330) and normal tissues (n=32) analyzed using the TCGA-OSCC data. (B) Heatmap of the correlation of 22 potential upstream transcription factors with *SOAT1*. The asterisks represented the statistical p-value (*$p < 0.05$, **$p < 0.01$, ***$p < 0.001$).

Figure S6 (A) RT-qPCR and Western Blot were used to verify the efficiency of *ETS1* overexpression and knockdown, simultaneously inspected the expression of *SOAT1* after altering *ETS1* expression. (B) Prediction of *ETS1* binding sites in the promoter region of *SOAT1*. The asterisks represented the statistical p-value (*$p < 0.05$, **$p < 0.01$, ***$p < 0.001$, ****$p < 0.0001$).

Figure S7 (A) RT-qPCR was used to determine the expression levels of *ETS1* in OSCC and paracancerous tissues (n=30). (B) Western Blot explores the effects of different concentrations of Polyphyllin I on the expression of *ETS1* and *SOAT1* in Cal27 cells after 48 h of treatment. (C) Western Blot was designed to test for changes in the expression of *ETS1* and *SOAT1* in PPI-treated Vector/oeETS1 Cal27 cells for 48 h. (D-E) IHC analysis of *ETS1* and *SOAT1* in 30 pairs of clinical samples of OSCC paired with adjacent tissue. (F-G) The expression levels of downstream lipid metabolism genes in OSCC cells with *ETS1*-overexpressing were checked using RT-qPCR and Western Blot. The asterisks represented the statistical p-value (*$p < 0.05$, **$p < 0.01$, ***$p < 0.001$, ****$p < 0.0001$).

Figure S8 Schematic diagram of nude mice treatment.
